# Bio‐inspired Catalyst‐Modified Photocathode for Bias‐Free Photoelectrochemical NADH Regeneration

**DOI:** 10.1002/advs.202413668

**Published:** 2024-12-30

**Authors:** Ziqi Zhao, Yizhou Wu, Chang Liu, Yingzheng Li, Chen Gong, Hongxia Ning, Peili Zhang, Fei Li, Licheng Sun, Fusheng Li

**Affiliations:** ^1^ State Key Laboratory of Fine Chemicals Frontier Science Center for Smart Materials Dalian University of Technology Dalian Liaoning 116024 China; ^2^ Center of Artificial Photosynthesis for Solar Fuels, School of Science Westlake University Hangzhou 310024 China

**Keywords:** photosynthesis, NADH regeneration, photocathode, bio‐inspired, catalyst‐modification

## Abstract

Cofactors such as nicotinamide adenine dinucleotide (NADH) and its phosphorylated form (NADPH) play a crucial role in natural enzyme‐catalyzed reactions for the synthesis of chemicals. However, the stoichiometric supply of NADH for artificial synthetic processes is uneconomical. Here, inspired by the process of cofactor NADPH regeneration in photosystem I (PSI), catalyst‐modified photocathodes are constructed on the surface of polythiophene‐based semiconductors (**PTTH**) via self‐assembly for photoelectrochemical catalytic NADH regeneration. With the assistance of viologen (**vi^2+^**) electron transfer mediators (similar function as Ferredoxin in PSI) linked to the [Rh(Cp^*^)(bpy)] catalyst, the **Rh‐vi^2+^@PTTH** photocathode exhibits higher photocurrent density (−665 µA cm^−2^) with a high apparent turnover frequency (TOF, 168.4 h^−1^) under a relatively positive potential (0.0 V vs RHE). In addition, through holistic functional mimics of the photosystem, a tandem photoelectrochemical cell is constructed by assembling a **CoPi@BiVO_4_** photoanode (artificial photosystem II, PSII) with the **Rh‐vi^2+^@PTTH** photocathode. This system achieves a production rate of 42.5 µm h^−1^ cm^−2^ and a TOF of 179.3 h^−1^ without an externally applied bias for NADH regeneration. The photo‐generated NADH is directly employed to assist glutamate dehydrogenase (GDH) in the catalytic conversion of α‐ketoglutarate to L‐glutamate. This study presents a novel strategic approach for constructing bias‐free photoelectrochemical NADH regeneration systems.

## Graphical abstract

Inspired by the structure and function of the thylakoid membrane and electron transport mediator function of Ferredoxin, a catalyst‐immobilized photocathode is constructed by self‐assembly of the aliphatic chain substituted [Rh(Cp^*^)(bpy)] molecules (**Rh‐vi^2+^**) and aliphatic chain decorated polythiophene semiconductors (**PTTH**), which can be coupled with a CoPi@BiVO_4_ photoanode resulting a bias‐free **CoPi@BiVO_4_||Rh‐vi^2+^@PTTH** tandem cell for efficient photoelectrochemical NADH regeneration.

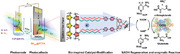

## 1 Introduction

In nature, green plants harvest and convert solar energy into chemical energy on the lipid bilayer thylakoid membrane through the highly efficient, photoinduced energy‐transfer cascade consisting of the Z‐scheme and the Calvin cycle.^[^ ^1^ ^]^ During natural photosynthesis, nicotinamide adenine dinucleotides, NAD(P)H and NAD(P)^+^, are the pivotal cofactor pairs that act as energy carriers in plenty of redox reactions involved with oxidoreductases.^[^ ^2^ ^]^ From the industrial point of view, oxidoreductases are widely used biocatalysts that enable many complex reactions with high reaction rates and excellent specificities under environmentally benign conditions.^[^ ^3^, ^4^ ^]^ However, broader applications of oxidoreductases are hindered by the requirement of stoichiometric supply of the expensive reducing equivalent, nicotinamide cofactor, NADH.^[^ ^5^ ^]^ Solar‐driven photoelectrochemical (PEC) cells could sustainably regenerate NADH cofactor from its oxidized form (NAD^+^), and continuously provide the reducing equivalent for oxidoreductases in a similar way to natural photosynthesis.^[^ ^6^ ^]^

PEC devices with photoanode||cathode^[^ ^7^, ^8^ ^]^ and anode||photocathode^[^ ^9^, ^10^, ^11^, ^12^ ^]^ configurations have been adopted to drive the Rh complex [Rh(Cp^*^)(bpy)] (a benchmark molecular catalyst, Cp^*^ = pentamethylcyclopentadienyl, bpy = 2,2′‐bipyridyl) for enzymatic active 1,4‐NADH regeneration. However, the overall PEC reaction driven by a single light‐absorbing layer remains challenging. To improve the conversion efficiency of photogenerated charge carriers, a large external bias is commonly required to adjust the Fermi levels and the band‐bending of photoelectrodes, which will consume electric power and reduce the economics of PEC devices. To address this issue, the photovoltaic device (PV) has been integrated in series with a photoelectrode to provide the additional driving force for charge separation.^[^ ^13^, ^14^ ^]^ Despite the external bias being avoided in such a configuration, the complexity of the system in design and fabrication will raise the cost and technical challenges of development and deployment. Natural photosynthesis uses a two‐stage, double‐excitation process (PSII and PSI, known as a “Z‐scheme”) to drive the overall reaction. Inspired by this, artificial photosynthesis was achieved by coupling photoanode and photocathode in one PEC cell for NADH regeneration.^[^ ^6^, ^15^, ^16^, ^17^ ^]^ This configuration of dual‐absorber tandem systems (photoanode||photocathode) is becoming increasingly appealing for unbiased overall PEC reactions, due to its advantage in balancing the device complexity, cost, and efficiency.^[^ ^18^, ^19^ ^]^

For tandem systems, both outstanding photoanodes and photocathodes are important. Compared to the well‐developed photoanodes for water oxidation, the development of photocathodes for NADH regeneration is relatively slow. In most of the reported photocathode systems, the molecular [Rh(Cp^*^)(bpy)] catalyst was directly dissolved in the electrolyte. Due to the mismatched timescales between electron transfer and mass transfer, only a small fraction of the catalyst in the electrical double layer can promptly capture photogenerated electrons and then utilize them for NADH regeneration. The horrendous waste of photogenerated electrons and the noble metal‐containing catalyst decreases the efficiency and increases the cost of such systems. The dissolved catalyst also leads to compatibility issues with oxidoreductases and increases the cost of product separation and catalyst recycling. Recently, a [Rh(Cp^*^)(bpy)] immobilized Si photocathode was developed for NADH regeneration.^[^ ^20^ ^]^ However, the inherent negative onset potential of Si and the poor electron transfer at the inorganic‐organic interface restrict its combination with photoanodes. Therefore, it remains a great challenge to develop photocathodes with positive onset potential, effective catalyst modification, and efficient electron transfer for unbiased tandem PEC cells.

**Figure 1:**
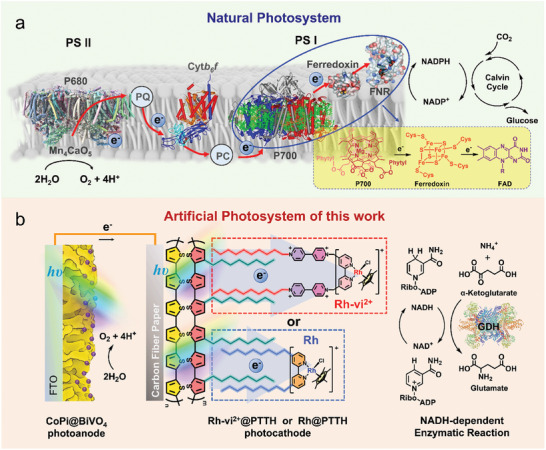
a) Schematic diagram of the electron transport chain from PSII to the PSI and Calvin cycle, and b) Schematic diagram of **CoPi@BiVO_4_||Rh‐vi^2+^@PTTH** or **CoPi@BiVO_4_||Rh@PTTH** tandem cells for light‐induced NADH regeneration and NADH‐dependent enzyme‐catalyzed reaction.

Natural photosynthesis elegantly addresses these issues via a fine‐tuned structure on the lipid bilayer. The well‐known electron transfer chain is demonstrated in **Figure** **1**a. Water oxidation is catalyzed by the Mn_4_CaO_5_ cluster of PSII driven by the excited P680 light‐harvesting antenna. Through an electron transportation chain, the electrons extracted from water are delivered to PSI, and then the light‐harvesting center P700 in PSI accepts electrons from PSII and then pumps them to a higher energy level by photoexcitation. The excited state P700^*^ is quenched by transferring the electron to the Ferredoxin mediator, which shuttles the electrons to the flavin adenine dinucleotide (FAD) in ferredoxin‐NADP^+^ reductase (FNR) for the hydrogenation of NADP^+^ to NADPH.

Herein, inspired by structure and electron transfer chain in natural photosynthesis, unbiased solar NADH regeneration PEC cells were constructed in a tandem configuration, consisting of a CoPi@BiVO_4_ photoanode and [Rh(Cp^*^)(bpy)] catalyst‐modified polythiophene‐based photocathodes (Figure 1b). The photocathodes were constructed by the self‐assembly of amphipathic [Rh(Cp^*^)(bpy)] catalysts (**Rh‐vi^2+^** and **Rh**) and aliphatic chain decorated polythiophene (**PTTH**) on carbon fiber, forming lipid bilayer membrane (LBM)‐like structures.^[^ ^21^, ^22^ ^]^ The integration of **Rh‐vi^2+^** and **Rh** with **PTTH** in a proximity configuration by the hydrophobic alkyl chain facilitates the electron transfer, leading to a remarkably positive shift of onset potential (130 mV in **Rh@PTTH** and 180 mV in **Rh‐vi^2+^@PTTH**) compared with **PTTH**. The viologen (**vi^2+^**) units of **Rh‐vi^2+^** act as electron transfer mediators similar to Ferredoxin in PSI, which facilitates electron transportation from the semiconductor layer to the catalyst center. With the assistance of **vi^2+^**, **Rh‐vi^2+^@PTTH** exhibited higher photocurrent density (−665 µA cm^−2^), NADH yield (16.0%), and Faradaic efficiency (FE, 32.1%) under a relatively positive potential (0.0 V vs RHE) than that of **Rh@PTTH** (−507 µA cm^−2^, yield: 11.4%, FE: 24.9%). The usage of [Rh(Cp^*^)(bpy)] was also promoted in both **Rh@PTTH** and **Rh‐vi^2+^@PTTH** with turnover frequencies (119.2 and 168.4 h^−1^, respectively) far exceeding the dissolved [Rh(Cp^*^)(bpy)] in reported works. Tandem PEC cells were constructed by combining the photocathodes with a cobalt phosphate‐decorated bismuth vanadate photoanode (**CoPi@BiVO_4_**). The **CoPi@BiVO_4_||Rh‐vi^2+^@PTTH** tandem cell demonstrates a yield of 25.5% and FE of 30.3% without bias potential.

## 2 Results and Discussion

Rhodium complexes **Rh**, **Rh‐vi** ^**2+**,^ and the alkane chain functionalized terthiophene (TTH_8C_) were synthesized and characterized (see “Synthetic Section,” Schemes S1–S7, and Figures S1–S13, Supporting Information). 2,2′:5′,2′“‐terthiophene (TTh) was electrochemically polymerized on carbon fiber paper (CP) through successive cyclic voltammetry scans.^[^ ^23^ ^]^ Then, TTH_8C_ was electrochemically polymerized on the surface of poly‐2,2′:5′,2′”‐terthiophene (pTH) films, resulting in **PTTH** films (Figure S14b, Supporting Information). The influence of polymerization cycles on TTh and TTH_8C_ was optimized (Note S1 and Figures S15–S23, Supporting Information). After this pretreatment, aliphatic chains were introduced on the surface of **PTTH**. Finally, by assembling the **Rh** and **Rh‐vi** ^**2+**^ complexes on the surface of alkyl chains‐modified PTTH via hydrophobic self‐assembly interaction,^[^ ^24^ ^]^ respectively, **Rh@PTTH** or **Rh‐vi** ^**2+**^ **@PTTH** electrodes were prepared (see Supporting Information for details).

**Figure 2:**
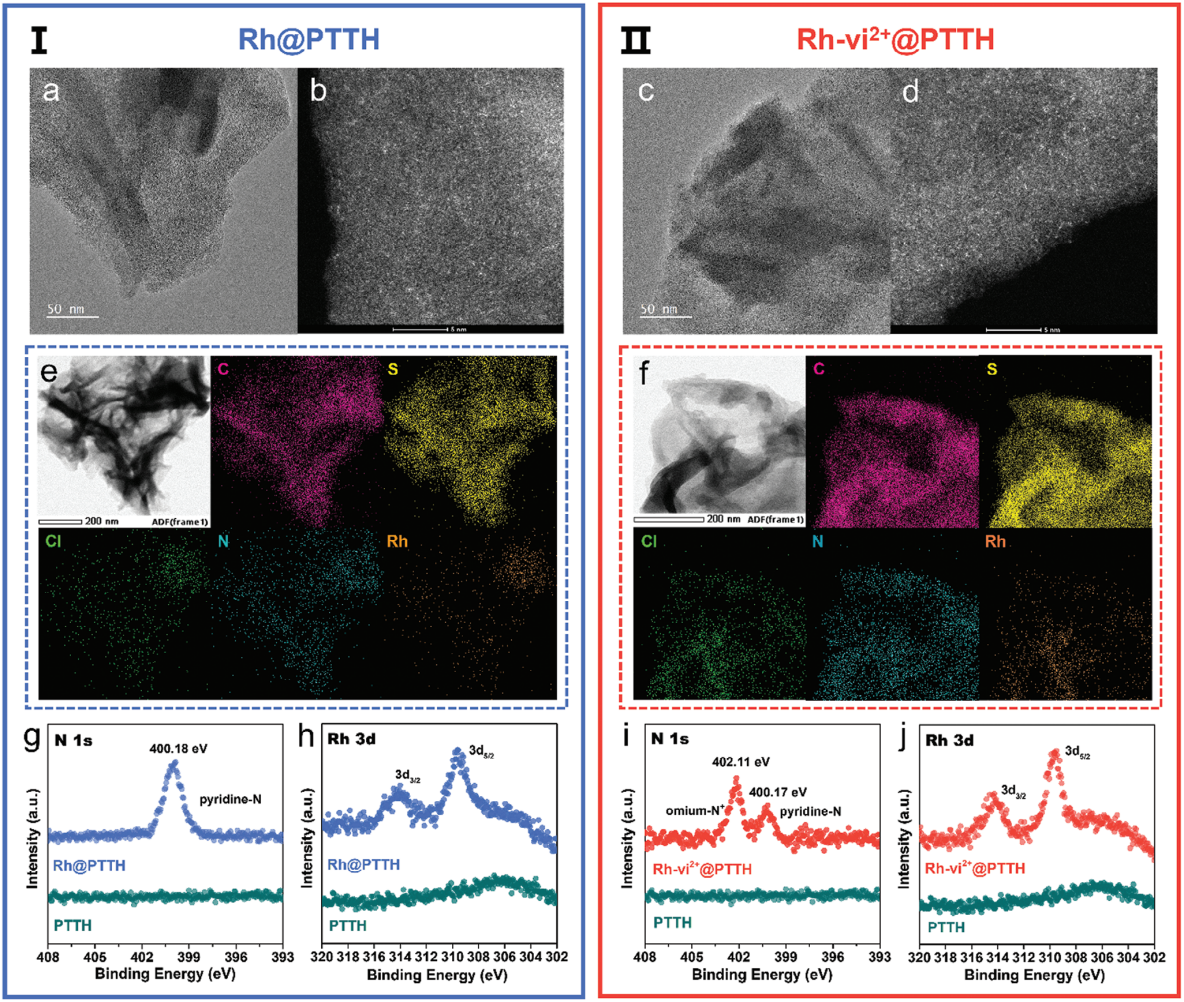
Characterization of two self‐assembled electrode systems for I: **Rh@PTTH** and II: **Rh‐vi^2+^@PTTH**. High‐resolution transmission electron microscopy (HRTEM) images of a) **Rh@PTTH** and c) **Rh‐vi^2+^@PTTH** films, AC‐HAADF‐STEM images of b) **Rh@PTTH** and d) **Rh‐vi^2+^@PTTH** films, bright spots correspond to monodisperse Rh atomic sites, HAADF‐STEM images and corresponding EDS elemental mappings of e) **Rh@PTTH** and f) **Rh‐vi^2+^@PTTH** surfaces, Elements detected: C, S, Cl, N, and Rh, High‐resolution XPS spectra of N 1s and Rh 3d of **Rh@PTTH** g,h) and **Rh‐vi^2+^@PTTH** (i,j).

The effective loading amounts of **Rh** and **Rh‐vi^2+^** on the surface of **PTTH** were determined to be 2.40 × 10^−7^ and 2.37 × 10^−7^ mol cm^−2^ by inductively coupled plasma atomic emission spectroscopy (ICP‐AES). As shown in Figure S24 (Supporting Information), minimal apparent macroscopic differences are observed among **PTTH**, **Rh@PTTH,** and **Rh‐vi^2+^@PTTH**. Atomic force microscope (AFM) images reveal that **Rh@PTTH** and **Rh‐vi^2+^@PTTH** have a similar root mean surface roughness of 10.6 and 11.9 nm in the selected area of 1 × 1 µm^−^2 (Figure S25, Supporting Information). Meanwhile, **Rh@PTTH** and **Rh‐vi^2+^@PTTH** display similar morphology to **PTTH** from scanning electron microscope (SEM) images (Figure S26, Supporting Information), indicating a molecular‐level modification of the self‐assembled process. Transmission electron microscope (TEM) images demonstrate a nanosheet‐agglomerated 3D nanoflower structure for **PTTH**. The self‐assembly of Rh catalysts did not change the microstructure of **PTTH** (**Figure** **2**a,c, Figure S27, Supporting Information). Aberration‐corrected high‐angle annular dark‐field scanning transmission electron microscopy (AC‐HAADF‐STEM) images show the existence of atomically dispersed Rh atoms (Figure 2b,d) on the surface of **Rh@PTTH** and **Rh‐vi^2+^@PTTH**. Corresponding energy‐dispersive spectroscopy (EDS) analysis demonstrates a uniform distribution of C, S, Cl, N, and Rh elements (Figure 2e,f). X‐ray photoelectron spectroscopy (XPS) survey spectra of **Rh@PTTH** and **Rh‐vi^2+^@PTTH** electrodes indicate the presence of the elements C, S, N, F, P, and Rh (Figure S28, Supporting Information). For **Rh@PTTH**, the N 1s peak located at 400.18 eV belongs to the N signal in pyridine for [Rh(Cp^*^)(bpy)] (Figure 2g). In Rh 3d spectrum, distinct characteristic peaks at 309.55 (3d_5/2_) and 314.25 eV (3d_3/2_) for Rh are also observed (Figure 2h).^[^ ^25^ ^]^ For **Rh‐vi^2+^@PTTH**, apart from the pyridine‐N signal at 400.17 eV, an additional peak is observed at 402.11 eV, which belongs to the N in **vi^2+^** (Figure 2i).^[^ ^26^ ^]^ The slight shift of Rh 3d_5/2_ (309.69 eV) and 3d_3/2_ (314.38 eV) toward a higher binding energy is attributed to the electron‐withdrawing property of **vi^2+^** (Figure 2j).^[^ ^23^ ^]^ Notably, the XPS signals of Rh and N in **Rh@PTTH** and **Rh‐vi^2+^@PTTH** electrodes are consistent with signals of molecular **Rh** and **Rh‐vi^2+^**, respectively (Figure S29 and Table S1, Supporting Information). The above characterizations indicated that both **Rh** and **Rh‐vi^2+^** have been successfully immobilized on the surface of **PTTH** with minimal changes occurring in the chemical environment of the catalysts.

The band structure of **PTTH** was measured by combining UV–vis absorbance spectra and UV photoemission spectroscopy (UPS) (Figure S30, Supporting Information). The band gap of **PTTH** is determined as 2.00 eV, with a valence band minimum (VBM) of 1.28 V versus RHE, and a conduction band maximum (CBM) of −0.72 V versus RHE (calculated by Equations S1 and S2, Supporting Information). Cyclic voltammetry (CV) and differential pulse voltammetry (DPV) analyses were carried out for **Rh**, **Rh‐vi^2+^**, and the ligand of **Rh‐vi^2+^** (**vi^2+^‐ligand**). As shown in Figure S31a,b (Supporting Information), reduction peaks at −0.08 and −0.07 V versus RHE observed for **Rh** and **Rh‐vi^2+^**, respectively, belong to the irreversible reduction of Rh^III^ to Rh^I^ in [Rh(Cp^*^)(bpy)].^[^ ^4^ ^]^ Meanwhile, a reversible redox peak of the **vi^+•^/vi°**a couple for the **vi^2+^‐ligand** at −0.16 V versus RHE is observed (Figure S31c, Supporting Information), consistent with that in **Rh‐vi^2+^**. The potential of the redox peak for **vi^2+^** units in **Rh‐vi^2+^** is more negative than the potential for Rh‐hydride formation. Accordingly, the energy‐band diagram of **PTTH**, **Rh**, and **Rh‐vi^2+^** is displayed in Figure S32 (Supporting Information). Specifically, the PTTH is excited by light to produce charge separation and then photogenerated electrons are injected into the conduction band of PTTH. The CBM of **PTTH** indeed provides sufficient driving force for **Rh** and **Rh‐vi^2+^** to catalyze the regeneration of NADH. However, there is a large energy cliff between the CB of **PTTH** and the reduction potential of the [Rh(Cp^*^)(bpy)], electrons need to overcome this energy barrier to avoid serious electron recombination and enhance the catalytic reaction on the electrode surface. Given the appropriate redox potential of **vi^+•^/vi°** couple in **Rh‐vi^2+^**, the electrons at the CB of **PTTH** can be received and then provided to the Rh center by viologen radicals. Therefore, the interfacial electron transfer between **PTTH** and [Rh(Cp*)(bpy)] could be further promoted in **Rh‐vi^2+^@PTTH** with the regulation of **vi^2+^** units.

**Figure 3:**
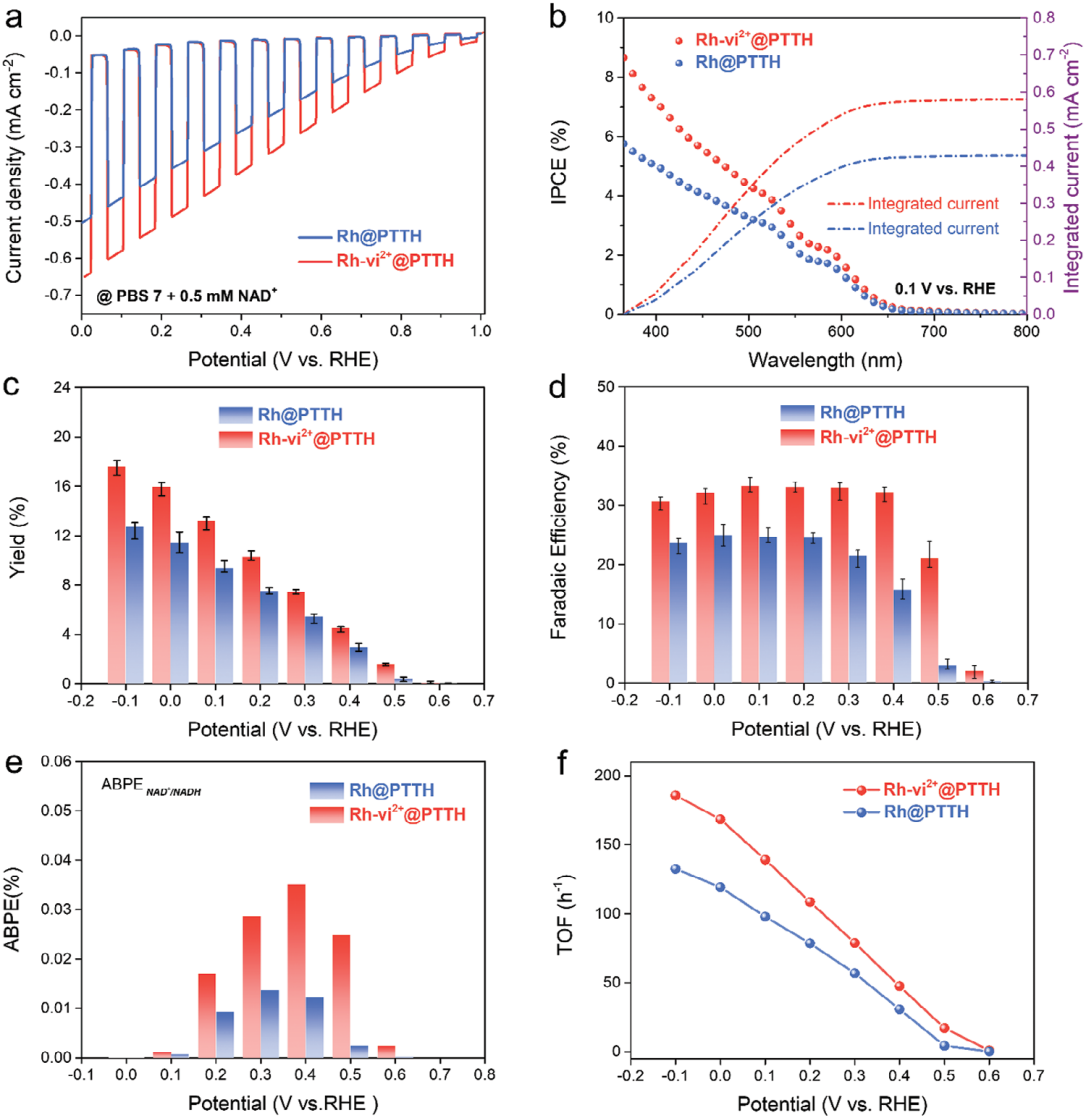
a) LSV curves of **Rh@PTTH** and **Rh‐vi^2+^@PTTH** photocathodes in 0.1 m PBS 7 + 0.5 mm NAD^+^ (Under chopped illumination). b) Incident‐photon‐to‐current efficiencies (IPCEs) of **Rh@PTTH** and **Rh‐vi^2+^@PTTH** at 0.1 V versus RHE under a monochromatic light at different wavelengths. Dash‐dot lines stand for respective integrating currents. Comparison between **Rh@PTTH** and **Rh‐vi^2+^@PTTH** systems NADH regeneration of c) NADH yield and d) Faradaic efficiency (error bar shows the maximum, minimum, and median values). e) Applied bias photon‐to‐current efficiencies (ABPEs) of **Rh@PTTH** and **Rh‐vi^2+^@PTTH** for NADH regeneration converted from the LSV curves and corresponding FEs at a certain potential. f) TOFs of **Rh@PTTH** and **Rh‐vi^2+^@PTTH** photocathodes at different potentials.

The basic PEC performances of the prepared electrodes were evaluated in an H‐type cell separated containing a common concentration of NAD^+^ (0.5 mm) used in PEC reaction by a proton exchange membrane (Nafion117). **PTTH** electrode exhibits a photocurrent density of −407 µA cm^−2^ at 0.0 V versus RHE with the presence of NAD^+^, which is higher than that of the control group without NAD^+^. Because the polythiophene‐based organic materials are excellent photoresponsive p‐type semiconductors, the photocurrent response of the blank control group may be contributed by the proton reduction process of water.^[^ ^27^, ^28^ ^]^ Concurrently, a non‐negligible dark current is observed for **PTTH** regardless of the presence of NAD^+^ (Figure S33a,b, Supporting Information). The photocurrent density of **Rh@PTTH** and **Rh‐vi^2+^@PTTH** photocathodes increases to −507 and −665 µA cm^−2^ at 0.0 V versus RHE with the reduced dark currents at the negative potential, respectively, after the immobilization of **Rh** and **Rh‐vi^2+^** catalysts (**Figure** **3**a). Notably, both photocathodes present weak transient spikes, indicating a fast photo response.

The onset potentials of **Rh@PTTH** and **Rh‐vi^2+^@PTTH** positively shifted to 0.95 and 1.0 V compared to **PTTH** (0.82 V vs RHE), indicating a promoted charge separation after the introduction of **Rh** and **Rh‐vi^2+^**. In addition, the photocurrents of **Rh@PTTH** and **Rh‐vi^2+^@PTTH** both increase after the addition of NAD^+^ (Figure S34a,b, Supporting Information), indicating that the hydrogenation of NAD^+^ promotes the recycling of **Rh** and **Rh‐vi^2+^** catalysts. In the existence of NAD^+^, the phenomena of an obvious increase in photocurrent and positive shift in onset potential are consistent with those of the reported polythiophene‐based photoelectrodes.^[^ ^11^ ^]^ The photoelectrode incident‐photon‐to‐current efficiency (IPCE) of **Rh‐vi^2+^@PTTH** is constantly greater than that of **Rh@PTTH** at a potential of 0.1 V versus RHE (Figure 3b). A maximum IPCE of 8.65% at 365 nm in **Rh‐vi^2+^@PTTH** is obtained while that of **Rh@PTTH** is 5.78%. The integrating current of **Rh@PTTH** and **Rh‐vi^2+^@PTTH** is calculated to be 427 and 578 µA cm^−2^ by integrating the corresponding IPCE curves over the solar irradiation (ASTM G173‐03) spectrum (Equation S3, Supporting Information), which are consistent with photocurrents in PEC measurements.

To evaluate the NADH regeneration rate, characteristic UV–vis absorption of commercial 1,4‐NADH at 340 nm was measured to establish the calibration curve (Figure S36, Supporting Information).^[^ ^29^, ^30^ ^]^ The effects of applied potential on NADH production by **Rh@PTTH** and **Rh‐vi^2+^@PTTH** were also explored. The absorbance at 340 nm of reaction solutions rises with the increase of applied potential after PEC measurements (Figure S37a–d, Supporting Information). The concentration of generated NADH was calculated according to Equation S4 and Figure S37e,f (Supporting Information). In the potential range of 1.0–0.7 V, no NADH was detected although there was a significant increase in the photocurrent, which could be attributed to the several indirect processes on the electrode.^[^ ^11^ ^]^ When the potential was lower than 0.6 V, regenerated NADH began to be observed. The NADH production gradually increased as the voltage became more negative. The highest yields of **Rh@PTTH** and **Rh‐vi^2+^@PTTH** are 12.7% and 17.5% at −0.1 V versus RHE in the monitored voltage range, respectively (Figure 3c, Equation S5, Supporting Information). Nevertheless, considering the non‐negligible dark current at this potential, it is more reasonable to investigate the yields (11.4% and 16.0%) at 0.0 V. Briefly, the NADH yield of the self‐assembly system was successfully improved by optimizing the photocurrent, selecting the appropriate potential, and regulating the electron transfer process. In addition, the NAD^+^ reduction ability of pristine **PTTH** was also evaluated in the absence of catalyst. As shown in Figure S38 (Supporting Information), compared with the **Rh** or **Rh‐vi^2+^** catalyst‐modified photocathodes, the NAD^+^ conversation yield of **PTTH** is much slower (1.8%) and accompanied by a deficient selectivity of 1,4‐NADH (27.4%). The results confirmed that the selective 1,4‐NADH regeneration is almost entirely contributed by the [Rh(Cp^*^)(bpy)] catalyst rather than the **PTTH** semiconductor itself. Because the UV–vis method is unable to distinguish 1,4‐NADH from the by‐products of NAD_2_ dimers or 1,6‐NADH,^[^ ^31^ ^] 1^H nuclear magnetic resonance (NMR) spectroscopy was performed to identify the enzymatic active 1.4‐NADH (Figure S39, Supporting Information).^[^ ^32^, ^33^ ^]^ The Faradaic efficiency (FE) of **Rh‐vi^2+^@PTTH** reaches a platform at 0.4 V versus RHE (higher than 32%, Figure 3d, Equation S6, Supporting Information). In contrast, **Rh@PTTH** reaches the platform at a more negative applied potential of 0.2 V versus RHE (less than 25%). Herein, **vi^2+^** units in **Rh‐vi^2+^@PTTH** successfully simulate the function of Ferredoxin to improve electron transfer, resulting in the improvement of FE. However, the FEs of immobilized Rh‐catalyzed NADH regeneration PEC systems still have room for improvement compared with those of reported homogeneous NADH regeneration systems. The less prominent values of FEs may be attributed to insufficient Rh catalytic site, competitive reactions such as HER, and self‐photo corrosion.

The applied bias photon‐to‐product efficiency (ABPE) was obtained by converting the LSV curves and FEs at a certain potential according to Figure 3d, Figure S35, and Equation S7 (Supporting Information) (please note: ABPE_NAD_^+^_/NADH_ is not the half‐cell of light conversion efficiency). As the bar chart is shown in Figure 3e, the maximum ABPE of **Rh@PTTH** and **Rh‐vi^2+^@PTTH** photocathodes is determined to be 0.014% at ≈0.3 V and 0.035% at ≈0.4 V versus RHE, respectively. The more positive potential and larger maximum ABPE of **Rh‐vi^2+^@PTTH** indicate that the **Rh‐vi^2+^@PTTH** is more effective in driving the photogenerated electron to participate in NADH regeneration. Accordingly, the turnover frequencies (TOFs) of the **Rh** and **Rh‐vi^2+^** on **PTTH** were estimated (Figure 3f, Equation S8, Supporting Information). The TOF of **Rh‐vi^2+^** is 168.4 h^−1^ at 0.0 V versus RHE, which is 1.4‐fold of **Rh** (119.2 h^−1^) on the surface of **PTTH** semiconductor. At a more positive applied potential of 0.5 V versus RHE, the TOF of **Rh‐vi^2+^** (16.9 h^−1^) is ≈4‐fold of **Rh** (4.3 h^−1^). The higher photocurrent, IPCE, conversion yield, and FE of **Rh‐vi^2+^@PTTH**, as well as the higher TOF of **Rh‐vi^2+^** reveal that the incorporation of **vi^2+^** units with the [Rh(Cp^*^)(bpy)] catalyst significantly promotes the photon‐to‐chemical conversion in NADH regeneration process. Table S2 (Supporting Information) lists the performance of **Rh@PTTH** and **Rh‐vi^2+^@PTTH** in the current work and previously reported systems for light‐involved NADH regeneration, including photocathodes and photocatalysts through PEC or photocatalytic regeneration routes. This comparison clearly demonstrates that the TOFs of [Rh(Cp^*^)(bpy)] in our catalyst‐modified photocathodes are much higher than those of photocathode or photocatalyst systems that dissolved [Rh(Cp^*^)(bpy)] in electrolytes. Meanwhile, considering the relatively low loading capacity of [Rh(Cp^*^)(bpy)] on the photocathodes, our immobilization strategy could dramatically reduce the cost of the system.

Further characterizations of **Rh@PTTH** and **Rh‐vi^2+^@PTTH** after PEC measurements were performed. The morphology of electrodes is barely changed with Rh complexes maintained on the surface of photocathodes after 2 h PEC measurements (Figures S40 and S41, Supporting Information). The amounts of **Rh** or **Rh‐vi^2+^** on the photocathodes after photoelectrolysis were determined to be 2.35 × 10^−7^ and 2.28 × 10^−7^ mol cm^−2^ by ICP‐AES, almost equal to the amounts before PEC measurements. Meanwhile, XPS spectra of the Rh 3d of **Rh@PTTH** or **Rh‐vi^2+^@PTTH** after PEC measurements are consistent with those of the photocathodes before measurements (Figure S42, Supporting Information). These characterizations indicate the considerable stability of the [Rh(Cp^*^)(bpy)] immobilized photocathodes prepared by the self‐assembly approach.

To investigate the role of **vi^2+^** units in **Rh‐vi^2+^@PTTH** during PEC NADH regeneration, photoelectrochemical impedance spectroscopy (PEIS) was adopted to explore the charge transfer property between the photocathodes and electrolyte interface. As shown in Figure S43a (Supporting Information), the PEIS Nyquist plots of **PTTH**, **Rh@PTTH**, and **Rh‐vi^2+^@PTTH** were recorded under continued illumination at 0.2 V versus RHE. The two semicircles of curves are fitted according to a typical equivalent circuit including series resistance (*R*_s_) and two *RC* elements in series (*RC*: resistors in parallel with capacitors) (Figure S43b, Supporting Information).^[^ ^34^ ^]^ The relevant fitting curves and parameters are shown in Figure S44 and Table S3 (Supporting Information). In the low‐frequency region, the charge transfer resistance (*R_ct_*), parallel with the Helmholtz capacitance (*C_h_*), reflects charge transfer information at the interface. In the high‐frequency region, the space‐charge resistance (*R_sc_*) in parallel with the space‐charge region capacitance (*C_sc_*) represents the bulk charge transport case in the semiconductor. From the Nyquist plots, charge transfer behavior can be intuitively embodied via the diameter magnitude of each semicircle.^[^ ^35^ ^]^ According to the fitting parameters, **PTTH** demonstrates the largest semicircle with the highest *R_ct_* (4074 Ω), which is attributed to the relatively slow reaction kinetics of **PTTH** itself. In contrast, **Rh@PTTH** and **Rh‐vi^2+^@PTTH** both display smaller semicircles, indicating that [Rh(Cp^*^)(bpy)] sites could facilitate interfacial charge transfer between **PTTH** and NAD^+^. Meanwhile, **Rh‐vi^2+^@PTTH** has a lower *R_ct_* and a higher *C_h_* (2305 Ω and 21.6 µF) than **Rh@PTTH** (3100 Ω and 19.1 µF), indicating a better charge transfer ability. However, the *R_sc_* and *C_sc_* for **Rh‐vi^2+^@PTTH** (232.5 Ω, 24.5 µF) are close to that of **Rh@PTTH** (228.6 Ω, 25.2 µF), suggesting a similar charge transfer behavior in the bulk phase of the **PTTH** semiconductor.

**Figure 4:**
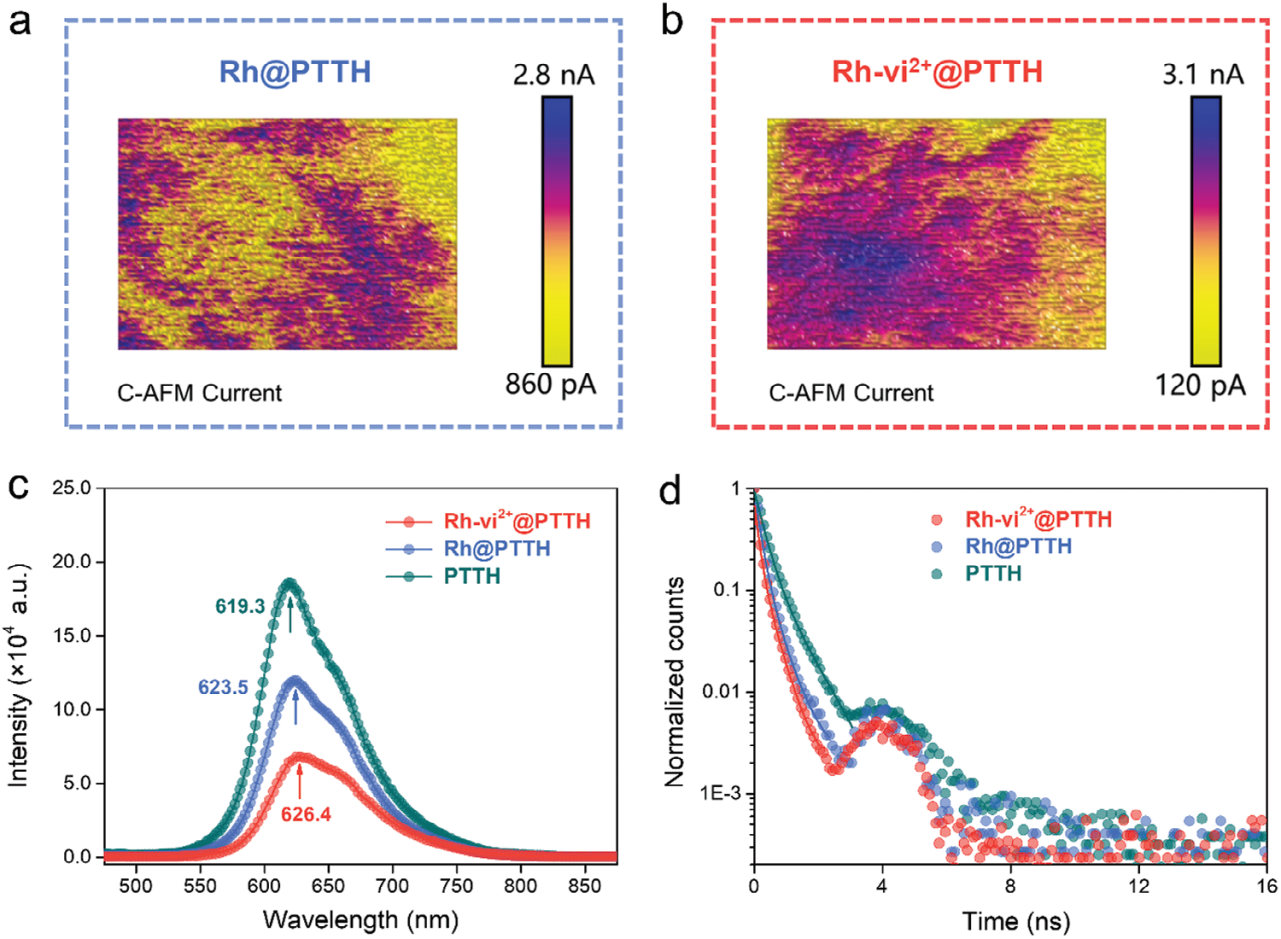
2D C‐AFM images of surface current distribution for a) **Rh@PTTH** and b) **Rh‐vi^2+^@PTTH** film. Photogenerated electrons transfer properties of **PTTH**, **Rh@PTTH**, and **Rh‐vi^2+^@PTTH** semiconductor films: c) Steady‐state photoluminescence (PL) spectra and d) Time‐resolved photoluminescence decay spectra (scatters) with corresponding fitting curves (lines).

Meanwhile, the conductivity of **Rh@PTTH** and **Rh‐vi^2+^@PTTH** was evaluated by a four‐probe method. As listed in Table S4 (Supporting Information), the film resistivity of **Rh@PTTH** and **Rh‐vi^2+^@PTTH** is determined to be ≈3.4 and 3.2 mΩ·cm (Equation S9, Supporting Information), respectively. The visible difference in film resistivity suggests that the conductivity of **Rh‐vi^2+^@PTTH** is slightly higher than that of **Rh@PTTH**. From a macro perspective, the similar resistivity of electrodes matched well with the result of equal values for *R_sc_*. Furthermore, a conductive atomic force microscope (C‐AFM) test was executed to assess the electrical properties and carrier transport properties of the **Rh@PTTH** and **Rh‐vi^2+^@PTTH** surface from a microscopic perspective. As shown in **Figure** **4**a,b, the surface current distribution of **Rh@PTTH** situates uniformly at a low level owning a current span of 2.0 nA. On the contrary, **Rh‐vi^2+^@PTTH** presents a much broader signal range with a current span interval of 3.0 nA. The broader uniform distribution of high current levels and larger current spans demonstrates better carrier transport in **Rh‐vi^2+^@PTTH**, which is matched with the result of the resistivity test. The promoted conductivity of **Rh‐vi^2+^@PTTH** arises from the electron shuttle property of **vi^2+^** units, which endows an additional carrier transport pathway in **Rh‐vi^2+^@PTTH** compared to **Rh@PTTH**.^[^ ^36^, ^37^ ^]^ Photoluminescence (PL) emission spectra could further clarify the charge separation and recombination process. As shown in Figure 4c, the maximum fluorescence emission intensity is observed for **PTTH** due to the fast recombination of photogenerated electron‐hole pairs. After the introduction of **Rh** or **Rh‐vi^2+^**, the fluorescence intensity of **PTTH** is significantly reduced owing to the rapid extraction of photogenerated electrons by **Rh** or **Rh‐vi^2+^**.^[^ ^38^, ^39^ ^]^ **Rh‐vi^2+^@PTTH** exhibits a lower fluorescence intensity than **Rh@PTTH**, which also confirms the role of the **vi^2+^** units as the electron mediator. Time‐resolved PL measurements were then carried out to understand charge transfer dynamics. The time‐resolved PL decay curves were fitted with bi‐exponential functions (Equations. S10 and S11, Supporting Information). As shown in Figure 4d, the fitting average fluorescent lifetime (***τ_average_***) of **PTTH** (0.522 ns) is ≈2‐fold longer than the other two photocathodes (Table S5, Supporting Information), revealing the roles of **Rh** or **Rh‐vi^2+^** as electron capturers to accelerate fluorescence decay.^[^ ^29^, ^39^ ^]^ **Rh‐vi^2+^@PTTH** demonstrates a smaller fluorescent lifetime (0.25 ns) than that of **Rh@PTTH** (0.29 ns), indicating a better ability in the extraction of photo‐generated electrons of **Rh‐vi^2+^**.^[^ ^40^ ^]^ The above analysis confirms the roles of **vi^2+^** in photoelectron extraction from **PTTH** and electron shuttling from **PTTH** to the [Rh(Cp^*^)(bpy)] center, which eventually accelerates the regeneration of NADH. The **vi^2+^** units of **Rh‐vi^2+^** act as electron transfer mediators similar to the function of Ferredoxin in PSI, which facilitates electron transfer events between the excited state P700^*^ and the flavin adenine dinucleotide in ferredoxin‐NADP^+^ reductase.

**Figure 5:**
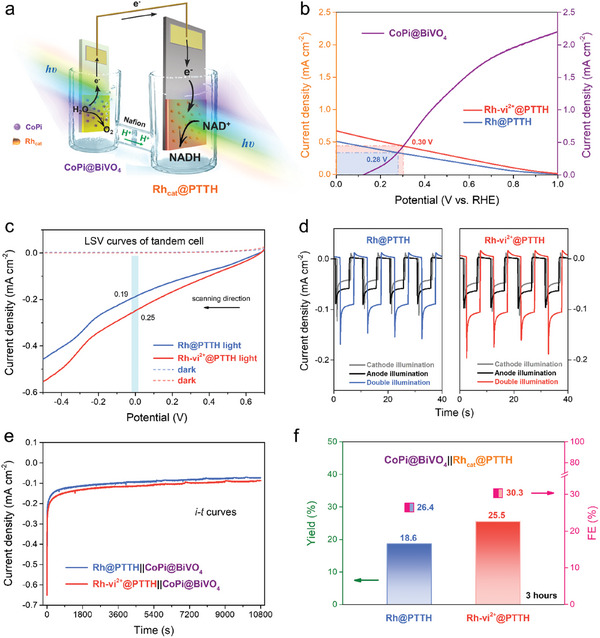
a) Schematic diagram of the unbiased tandem PEC cell, containing the **PTTH‐**based photocathode and **CoPi@BiVO_4_** photoanode. b) The current‐potential intersection of **Rh@PTTH** or **Rh‐vi^2+^@PTTH** with **CoPi@BiVO_4_**. c) LSV measurements of tandem PEC cells under both‐side illumination. d) Transient current responses of different sides chopped illumination for unbiased tandem PEC cells. e) *I–t* curves of unbiased tandem PEC cells. f) NADH yield and FE of unbiased tandem PEC cells.

With efficient NADH regeneration photocathodes in hand, tandem PEC cells were fabricated with the photoanode||photocathode configuration. Bismuth vanadate (BiVO_4_) based photoanodes exhibit excellent water oxidation performance and suitable band structures for the construction of Z‐scheme with polythiophene semiconductors.^[^ ^41^, ^42^ ^]^ Therefore, **Rh‐vi^2+^@PTTH** or **Rh@PTTH** photocathodes were coupled with a **CoPi@BiVO_4_** photoanode to perform the unbiased PEC NADH regeneration (**Figures** **5**a, S45, Supporting Information). As shown in Figure S46 (Supporting Information), three‐electrode PEC analysis of the **CoPi@BiVO_4_** photoanode displays an onset potential of 0.14 V versus RHE and a current density of 2.2 mA cm^−2^ at 1.0 V versus RHE. To estimate the operating current of the tandem device, a plot overlaying the *i*–*V* curves of the **CoPi@BiVO_4_** photoanode and **Rh@PTTH** or **Rh‐vi^2+^@PTTH** photocathodes is constructed (Figure 5b). **Rh@PTTH** and **Rh‐vi^2+^@PTTH** photocathodes reveal intersectional photocurrents of ≈340 and 440 µA cm^−2^ at 0.28 and 0.30 V, respectively, with the curve of **CoPi@BiVO_4_** photoanode, demonstrating the feasibility of unbiased PEC NADH regeneration in the tandem structure. As shown in Figure 5c, photocurrents of 190 and 250 µA cm^−2^ are obtained by the **CoPi@BiVO_4_||Rh@PTTH** and **CoPi@BiVO_4_||Rh‐vi^2+^@PTTH** tandem PEC cells, respectively, when the potential between photocathodes and photoanode was 0 V. The measured actual photocurrents of two bias‐free tandem cells are smaller than the theoretically predicted value in Figure 5b, which may be caused by the ohmic loss of the cationic pathway in the Nafion membrane and electrolyte.^[^ ^6^, ^43^ ^]^ To further understand the roles of the photoanode and photocathode in tandem PEC systems, *i*–*t* curves of **CoPi@BiVO_4_||Rh@PTTH** and **CoPi@BiVO_4_||Rh‐vi^2+^@PTTH** PEC cells under different illumination patterns on the photoelectrodes (Figure S47, Supporting Information) were recorded in a bias‐free situation (Figure 5d). When both photoanode and photocathode are irradiated, the photocurrents are obviously higher than those of single‐side illumination. Meanwhile, stable state photocurrent ≈88 and 105 µA cm^−2^ can be obtained for **CoPi@BiVO_4_||Rh@PTTH** and **CoPi@BiVO_4_||Rh‐vi^2+^@PTTH**, respectively. Therefore, long‐term unbiased NADH regeneration reactions were performed (Figure **5** **e**). The *i*–*t* degradation behaviors in tandem systems were similar to the photocurrent decay trends of photocathodes alone at different constant potentials (Figure S37a,b, Supporting Information). The higher photocurrent of **CoPi@BiVO_4_||Rh‐vi^2+^@PTTH** than that of **CoPi@BiVO_4_||Rh@PTTH** observed at all stages, which is consistent with results in photocathode measurements, reconfirming the critical role of **vi^2+^** units as electron transfer mediators. According to the UV–vis absorption spectra of the cathode compartment (Figure S48, Supporting Information) and the charge passed through the electrode, the NADH yields and FEs of **CoPi@BiVO_4_||Rh@PTTH** and **CoPi@BiVO_4_||Rh‐vi^2+^@PTTH** were calculated (Figure 5f). Significantly higher conversion yield and FE of **CoPi@BiVO_4_||Rh‐vi^2+^@PTTH** (25.5%, 30.3%) are observed compared with those of **CoPi@BiVO_4_||Rh@PTTH** (18.6%, 26.4%). Furthermore, the TOF of **Rh‐vi^2+^** and **Rh** is 179.3 and 129.1 h^−1^, respectively, far exceeding the values reported in other NADH regeneration tandem systems (Table S2, Supporting Information). During the PEC reaction, the amount of NADH generated by **CoPi@BiVO_4_||Rh‐vi^2+^@PTTH** increased linearly with time, resulting in an average NADH regeneration rate of 42.5 µm h^−1^ cm^−2^ (Figure S49, Supporting Information).

The generated NADH by the unbiased **CoPi@BiVO_4_||Rh‐vi^2+^@PTTH** tandem cell was further applied for a model reaction, the conversion of α‐ketoglutarate to glutamate, under the assistance of GDH. The amount of α‐ketoglutarate and glutamate before and after the enzymatic reaction was measured by high‐performance liquid chromatography (HPLC) (Figure S50a,b, Supporting Information). As shown in Figure S50c (Supporting Information), the enzymatic reaction can not occur when **CoPi@BiVO_4_||Rh‐vi^2+^@PTTH** is placed in the dark without the photo‐generated NADH. On the contrary, the photo‐generated NADH originating from the above unbiased PEC reaction can drive GDH to convert α‐ketoglutarate to glutamate, with a glutamate production rate of 70.5 µm h^−1^ within 2 h. Simultaneously, NADH could be completely consumed after the enzymatic reaction (97.7%, Figure S50d, Supporting Information), substantiating that the photo‐generated NADH by **CoPi@BiVO_4_||Rh‐vi^2+^@PTTH** unbiased tandem cell was bioactive 1,4‐NADH.^[^ ^9^, ^11^ ^]^ The enzyme verification reveals the great potential of this catalyst‐immobilized photoelectrode‐based tandem cell in coupling with oxidoreductases for the synthesis of high‐value‐added chemicals.

## 3 Conclusion

Inspired by the structure and function of the thylakoid membrane and electron transport mediator function of Ferredoxin, catalyst‐immobilized photocathodes were constructed by self‐assembly of the aliphatic chain substituted [Rh(Cp^*^)(bpy)] molecules (**Rh** and **Rh‐vi^2+^**) and aliphatic chain decorated polythiophene semiconductors (**PTTH**). Both **Rh@PTTH** and **Rh‐vi^2+^@PTTH** photocathodes displayed outstanding performance for PEC NADH regeneration. Especially, when the viologen (**vi^2+^**) units were introduced as the electron transfer mediator between alkyl chains and the [Rh(Cp^*^)(bpy)] catalytic center, the **Rh‐vi^2+^@PTTH** photocathode exhibited an outstanding PEC performance with a great photocurrent density (−665 µA cm^−2^), a high NADH yield (16.0%), and a considerable FE (32.1%) at the relative positive potential 0.0 V versus RHE, accompanied by a remarkable TOF (168.4 h^−1^). The mechanistic study demonstrates that **vi^2+^** units act as electron transfer mediators that promote the photogenerated electrons transportation from the **PTTH** semiconductor to the [Rh(Cp^*^)(bpy)] catalytic center for NADH regeneration. Moreover, coupled with a **CoPi@BiVO_4_** photoanode, a bias‐free **CoPi@BiVO_4_||Rh‐vi^2+^@PTTH** tandem cell was successfully constructed for PEC NADH regeneration, resulting in a yield of 25.5% with FE of 30.3% and a production rate of 42.5 µm h^−1^ cm^−2^. The photo‐generated NADH could be employed to assist GDH in catalyzing the conversion of α‐ketoglutarate to L‐glutamate. This study may provide new inspiration for the design and construction of bias‐free photoelectrochemical NADH regeneration systems to produce a broader range of high‐value‐added chemicals by using catalyst‐modified photocathodes coupled with suitable photoanodes.

## Conflict of Interest

The authors declare no conflict of interest.

## Supporting information



Supporting Information

## Data Availability

The data that support the findings of this study are available from the corresponding author upon reasonable request.
