# Supplementary material for: Bio‐inspired Catalyst‐Modified Photocathode for Bias‐Free Photoelectrochemical NADH Regeneration
Source: Adv Sci (Weinh). 2024 Dec 30;12(8):2413668. doi: 10.1002/advs.202413668 (PMC11848594; doi:10.1002/advs.202413668)
Supplement: Supplementary file 1 — Supporting Information [file ADVS-12-2413668-s001.docx]

Supporting Information

Bio-inspired Catalyst-Modified Photocathode for Bias-Free Photoelectrochemical NADH Regeneration

*Ziqi Zhao,^a^ Yizhou Wu,^b^ Chang Liu,^a^ Yingzheng Li,^a^ Chen Gong,^a^ Hongxia Ning,^a^ Peili Zhang,^a^ Fei Li,^a^ Licheng Sun^a,b^ and Fusheng Li^*a^*

1. State Key Laboratory of Fine Chemicals, Frontier Science Center for Smart Materials, Dalian University of Technology, 116024 Dalian, Liaoning, China.
2. Center of Artificial Photosynthesis for Solar Fuels, School of Science, Westlake University, 310024 Hangzhou, China.

*Corresponding author: [*fusheng@dlut.edu.cn*](mailto:fusheng@dlut.edu.cn)

# Materials and Reagents

Potassium hexafluorophosphate (KPF_6_, 99%), anhydrous sodium perchlorate (NaClO_4_, 99%), anhydrous sodium sulfate (Na_2_SO_4_, 99%), sodium phosphate dibasic (Na_2_HPO_4_, 99%), sodium phosphate monobasic (NaH_2_PO_4_, 99.5%), potassium phosphate dibasic (K_2_HPO_4_, 99%), potassium phosphate monobasic (KH_2_PO_4_, 99%), α-ketoglutarate (C_5_H_6_O_5_, 98%), ammonium sulfate ((NH_4_)_2_SO_4_, 99.0%), bismuth nitrate pentahydrate (Bi(NO_3_)_3_·5H_2_O, 99.0 %), KI (99 %), vanadyl acetylacetonate (VO(acac)_2_, 99%), cobalt nitrate hexahydrate (Co(NO_3_)_2_·6H_2_O, 99.99%), potassium hydroxide (KOH, 99.9%), and boric acid (H_3_BO_3_, 99.8%) were purchased from *Aladdin*. Glutamate was purchased from *Adamas*. Glutamate dehydrogenase (GLDH, 250 units mg^−1^) was obtained from *Yuanye Biolog*. 4,4'-bipyridine (99%) was purchased from *TCI*. 2-bromo-3-octylthiophene (97%) was purchased from *Macklin*. 2,2':5',2''-terthiophene (99%), 4,4'-dinonyl-2,2'-bipyridine (98%), 4,4'-bis(bromomethyl)-2,2'-bipyridine (98%), 1-bromodecane (99%), Dichloro(pentamethylcyclopentadienyl)rhodium (III) dimer ((Cp*RhCl_2_)_2_, 98%), 2,5-dibromothiophene (98%), 1,3-Bis(diphenylphosphino)propane nickel chloride (Ni(dppp)Cl_2_, 98%) were purchased from *BidePharm*. β-Nicotinamide adenine dinucleotide hydrate (NAD^+^, 98%) and 1,4-Dihydronicotinamide adenine dinucleotide disodium salt (NADH, 98%) were purchased from *Sigma-Aldrich*. Carbon Fiber Paper (CP, TORAY TGP-H-060) was cut into a 1.0×2.5 cm^2^ rectangle for electro-polymerization. The CP substrates were cleaned with hydrochloric acid and alcohol by ultrasonic for 20 minutes before the experiment, successively. Proton exchange membrane (Nafion117, Dupont) was purchased from SINERO Technology. Hydrochloric acid (HCl, 37%) and Magnesium turning were purchased from local suppliers. Deionized water (18.2 MΩ·cm^−1^) was supplied by a Milli-Q system (Millipore, Direct-Q 3 UV). All reagents were commercially available and used as received. The organic solvents involved in the experiment (eg. MeOH, CH_3_CN, CH_2_Cl_2_, ether, n-hexane) were provided by local suppliers without further purification.

# Physical Characterization and Instruments

The morphology with corresponding elemental mappings of all as-prepared films was characterized by field emission scanning electron microscopy (JSM-7610F Plus, JEOL, operated at 5 and 10 kV) and field emission transmission electron microscopy (JEM-F200, JEOL). ^1^H-NMR and ^13^C-NMR spectra were obtained by Bruker AVANCE 500 or Bruker AVANCE NEO 600M Spectroscopy. at 298 K High-resolution and low-resolution mass spectrometry measurements were performed on a SynaptG2-Si HDMS (Waters™). The binding energy of relevant elements for surface compositions and the band diagrams of the electrode films were investigated using X-ray photoelectron spectroscopy (XPS) and ultraviolet photoelectron spectroscopy (UPS) on an ESCALAB Xi^+^ (Thermo Fisher Scientific™). The UV-vis absorption spectra of photocathodes were taken by a solid UV-visible spectrophotometer UV-3600 Plus (SHIMADZU). The UV-vis absorption spectra of all electrolytes after the reaction were determined by a liquid UV-visible spectrometer (Cary 60 UV-Vis, Agilent). Fourier transform infrared spectra (FT-IR) were measured by Nicolet iS50 (Thermo Fisher Scientific™). The vibration and rotation of chemical bands were investigated by a confocal and high-resolution Raman Spectrometer (LabRAM Odyssey, HORIBA Scientific). The specific surface areas of the semiconductor electrode films were estimated by the Brunauer-Emmett-Teller (BET) test on an ASAP 2460 Surface Area & Porosimeter. The water contact angle test of the semiconductor material surface was performed on the SZ-CAMC32 instrument. The thickness of the organic polymer films was detected by a stylus profilometer (Bruker, Dektak XT). The semiconductor film resistivity was measured by the four-point probe method (Hall 8800). Three-dimensional nano morphology and surface electrical properties of thin films were detected by atomic force microscope AFM (Dimension Icon, Bruker). The loading amount of the Rh catalyst was obtained by inductively coupled plasma-atomic emission spectroscopy (ICP-AES, AVIO 500 PerkinElmer™). The steady-state and time-resolved photoluminescence (PL) of the semiconductor electrode films were determined by the FLS1000 Photoluminescence Spectrometer. High-performance liquid chromatography (HPLC, 1260 II Agilent) was employed to detect the production of glutamate. The pH of the electrolyte was measured by the 914 pH/conductometer (Metrohm™).

# Synthetic Section

### A. Rh complex

#### Synthesis of Cp*Rh[4,4′-dinonyl-bpyCl]Cl (1)

**Rh** was synthesized according to our previous procedure with optimization.^1^

**Scheme S1***.* Synthetic routes of **1**.

Rh dimer molecule (Cp*RhCl_2_)_2_ (250 mg, 0.4 mmol) was first dispersed in 20 mL methanol and stirred for 10min, then 4,4′-dinonyl-2,2′-bipyridine (332 mg, 0.8 mmol) was slowly added in the dispersion. 5 mL of methanol was added to dilute the reaction. After 3 minutes of vigorous stirring at room temperature, the dispersion gradually turned to transparent and the white powder (4,4′-dinonyl-bpy) was completely dissolved after stirring for 5 hours. Finally, the clear orange solution was concentrated to 2 mL and was added 30 mL ether to obtain 575 mg yellow precipitate **1**, Yield: 99.1%. ^1^H NMR (600 MHz, Methanol-*d*_4_): δ 8.83 (d, *J* = 5.8 Hz, 2H), 8.47 (s, 2H), 7.70-7.71 (dd, *J* = 5.8, 1.7 Hz, 2H), 2.91 (t, *J* = 7.8 Hz, 4H), 1.79 (p, *J* = 7.5 Hz, 4H), 1.73 (s, 15H), 1.45-1.28 (m, 24H), 0.90 (t, *J* = 6.8 Hz, 6H).

**Figure S1**. ^1^H-NMR spectrum of **1**.

#### Synthesis of Cp*Rh[4,4′-dinonyl-bpyCl]PF_6_ (Rh)

**Scheme S2**. Synthetic routes of **Rh**.

Firstly, 570 mg of **1** was dissolved in a 4 mL mixture of methanol and water (1:1). Then excessive saturated aqueous potassium hexafluorophosphate solution (50 mL) was added. After thorough stirring overnight, the precipitate was filtered and collected to give 540 mg orange solid product **Rh**, Yield 82.2%. ^1^H NMR (500 MHz, Acetonitrile-*d*_3_): δ 8.70 (d, *J* = 5.7 Hz, 2H), 8.23 (s, 2H), 7.61 (dd, *J* = 5.8, 1.8 Hz, 2H), 2.85 (t, *J* = 7.7 Hz, 4H), 1.75 (p, *J* = 7.7 Hz, 4H), 1.65 (s, 15H), 1.40-1.26 (m, 24H), 0.88 (t, *J* = 7.1 Hz, 6H). ^13^C NMR (126 MHz, Acetonitrile-*d*_3_): δ 157.06, 153.93, 150.95, 127.97, 123.33, 96.72, 34.54, 31.32, 29.50, 28.88, 28.74, 28.70, 28.54, 22.09, 13.08, 7.85. HR-MS (ESI, m/z): calcd for 681.3202 [M-PF_6_^-^]^+^, found m/z =681.3412; calcd for 323.1603 [M-Cl-PF_6_]^2+^, found m/z =323.1869.

**Figure S2**. ^1^H-NMR spectrum of **Rh**.

**Figure S3**. ^13^C-NMR spectrum of **Rh**.

### B. Rh-vi^2+^ complex

#### Synthesis of 1-decyl-[4,4′-bipyridine]-1-ium bromide (2)

**Scheme S3**. Synthetic routes of **2**.

4,4′-bipyridine (5 g, 32 mmol) was dissolved in 40 mL acetonitrile, and 1-bromodecane (7.08 g, 32mmol) in 10mL acetonitrile was added dropwise slowly. The solution was refluxed with stirring at 50°C for 48 hours. The solution was filtered while hot to obtain the upper clear solution. The supernatant was concentrated to 10 mL by evaporation and then 50mL ether was added to give a yellowish precipitate. Finally, the precipitate was washed by ether (3×20 mL) and dried under vacuum to obtain a Mono-alkyl pyridinium salt product 10.2 g, Yield: 84.4%. ^1^H NMR (600 MHz, D_2_O): δ 8.85 (d, *J* = 6.8 Hz, 2H), 8.67 (d, *J* = 6.4 Hz, 2H), 8.30 (d, *J* = 6.9 Hz, 2H), 7.80 (d, *J* = 6.3 Hz, 2H), 4.54 (t, *J* = 7.3 Hz, 2H), 1.98-1.91 (p, *J* = 7.2 Hz, 2H), 1.27-1.09 (m, 14H), 0.71 (t, *J* = 6.5 Hz, 3H). ^13^C NMR (126 MHz, D_2_O): δ 153.80, 150.01, 144.73, 142.66, 126.00, 122.47, 61.69, 31.13, 30.34, 28.43, 28.33, 28.29, 27.88, 25.03, 21.98, 13.35. HR-MS (ESI, m/z): calcd for 297.2325 [M-Br]^+^, found m/z =297.2230.

**Figure S4**. ^1^H-NMR spectrum of **2**.

**Figure S5**. ^13^C-NMR spectrum of **2**.

#### Synthesis of 1′,1′′′-([2,2′-bipyridine]-4,4′-diylbis(methylene)) bis(1-decyl-[4,4′-bipyridine]-1,1′-diium) bromide (3)

**Scheme S4**. Synthetic routes of **3**.

According to literature,^2^ a triple excess of **2** (3.31 g, 8.7mmol) was dissolved in 50 mL DMF and heated at 90°C, then 4,4′-bis(bromomethyl)-2,2′-bipyridine (1 g, 2.9 mmol) dissolved in 20 mL hot DMF was slowly added by constant pressure funnel in 20 minutes. The mixture was stirred at 110°C for 72 h. After evaporating most of the DMF, the mixture was poured in 50 mL Et_2_O to obtain a large amount of light-yellow precipitate. The solid was filtered, washed three times with cold ether and dried in a vacuum, resulting in 2.45 g ligand molecule **3**, Yield: 76.5%. ^1^H NMR (600 MHz, D_2_O): δ 9.24 (d, *J* = 6.7 Hz, 4H), 9.10 (d, *J* = 6.5 Hz, 4H), 8.69 (d, *J* = 5.3 Hz, 2H), 8.61 (d, *J* = 6.4 Hz, 4H), 8.53 (d, *J* = 6.3 Hz, 4H), 8.14 (s, 2H), 7.53 (dd, *J* = 5.2, 1.9 Hz, 2H), 6.12 (s, 4H), 4.69 (t, *J* = 7.3 Hz, 4H), 2.04 (p, *J* = 7.4 Hz, 4H), 1.35-1.15 (m, 28H), 0.76(t, *J* = 6.7 Hz, 6H). ^13^C NMR (126 MHz, D_2_O ) δ 155.58, 151.15, 150.32, 149.66, 146.22, 145.53, 143.85, 127.54, 127.02, 123.94, 121.40, 63.12, 62.36, 31.12, 30.47, 28.45, 28.34, 28.31, 27.91, 25.07, 21.98, 13.34. MS (ESI, m/z): calcd for 194.14 [M-4Br]^4+^, found m/z =194.11; calcd for 258.52 [M-4Br-H]^3+^, found m/z =258.48; calcd for 468.19 [M-2Br]^2+^, found m/z =468.14.

**Figure S6**. ^1^H-NMR spectrum of **3**.

**Figure S7**. ^13^C-NMR spectrum of **3**.

#### Synthesis of Cp*Rh[4,4′-di(decyl-vi^2+^)-bpyCl]Cl (4)

**Scheme S5**. Synthetic routes of **4**.

The synthesis of **4** was similar to that of **1.** Ligand molecule **3** (882 mg, 0.8 mmol) dissolved in 4 mL MeOH was added dropwise to the suspension of Rh dimer molecule (Cp*RhCl_2_)_2_ (250 mg, 0.4 mmol) under stirring. Gradually the solution became clarified within 5 mins, followed by stirring overnight to obtain an orange transparent solution. The solution was concentrated to 2 mL and added into 30 mL ether to obtain a yellow solid product, Yield: 1.07 g (96%). ^1^H NMR (600 MHz, Methanol-*d*_4_): δ 9.58 (d, *J* = 6.4 Hz, 4H), 9.30 (d, *J* = 6.5 Hz, 4H), 9.07 (s, 2H), 9.04 (d, *J* = 5.8 Hz, 2H), 8.79 (d, *J* = 6.9 Hz, 4H), 8.73 (d, *J* = 6.4 Hz, 4H), 8.03 (dd, *J* = 5.9, 2.2 Hz, 2H), 6.35 (s, 4H), 4.75 (t, *J* = 7.6 Hz, 4H), 2.10 (p, *J* = 7.5 Hz, 4H), 1.79 (s, 15H), 1.46-1.26 (m, 28H), 0.89 (t, *J* = 6.9 Hz, 6H). ^13^C NMR (126 MHz, Methanol-*d*_4_): δ 156.03, 154.61, 152.47, 151.31, 148.08, 147.59, 147.14, 129.23, 128.61, 125.78, 99.60, 99.54, 63.42, 63.24, 33.06, 32.64, 30.64, 30.56, 30.43, 30.20, 27.29, 23.74, 14.45, 9.40. HR-MS (ESI, m/z): calcd for 364.4919 [M-3Br-2Cl-2H]^3+^, found m/z =364.4915

**Figure S8**. ^1^H-NMR spectrum of **4**.

Figure S9. ^13^C-NMR spectrum of 4.

#### Synthesis of Cp*Rh[4,4′-di(decyl-vi^2+^)-bpyCl]PF_6_ (Rh-vi^2+^)

**Scheme S6**. Synthetic routes of **Rh-vi^2+^**.

700 mg of **4** was dissolved in a 4 mL mixture of methanol and water (1:1), which was added in a potassium hexafluorophosphate saturated solution (60 mL) for anion exchange. An orange solid product **Rh-vi^2+^** (680 mg) was obtained, Yield: 77%. ^1^H NMR (500 MHz, Acetonitrile-*d*_3_): δ 9.08 (d, *J* = 6.4 Hz, 4H), 8.96-8.85 (m, 6H), 8.55 (s, 2H), 8.48 (d, *J* = 6.3 Hz, 4H), 8.40 (d, *J* = 6.3 Hz, 4H), 7.74 (d, *J* = 5.7 Hz, 2H), 6.05 (s, 4H), 4.62 (t, *J* = Hz, 4H), 2.02(p, 4H), 1.70 (s, 15H), 1.43-1.24 (m, 28H), 0.89 (t, *J* = 6.6 Hz, 6H). ^13^C NMR (101 MHz, Acetonitrile-*d*_3_) δ 155.28, 154.16, 152.33, 150.67, 147.38, 146.57, 146.47, 128.89, 128.30, 124.96, 99.20, 99.12, 63.35, 63.19, 32.55, 31.89, 30.12, 29.99, 29.94, 29.56, 26.52, 23.33, 14.32, 9.37. MS (ESI, m/z): calcd for 742.22 [M-2PF_6_^-^]^2+^, found m/z =742.16; calcd for 446.49 [M-3PF_6_^-^]^3+^, found m/z =446.45.

**Figure S10**. ^1^H-NMR spectrum of **Rh-vi^2+^**.

**Figure S11**. ^13^C-NMR spectrum of **Rh-vi^2+^**.

### C. Alkyl chains terthiophene derivative

#### Synthesis of 3,3′′-dioctyl-2,2′:5′,2′′-Terthiophene (TTH_8C_)

**Scheme S7***.* Synthetic routes of **TTH_8C_**.

3,3′′-dioctyl-2,2′:5′,2′′-Terthiophene (**TTH_8C_**) was synthesized according to literatures.^3-5^ A solution of 2-bromo-3-octylthiophene (8.00 g, 29.04 mmol) in diethyl ether (50 mL) was added to a suspension of Mg turnings (0.7 g, 29.04 mmol) in ether (20 mL) under N_2_. The mixture was sonicated until the entire disappearance of the Mg turnings. The solution of above Grignard reagent was then added dropwise by cannula to a mixture of 2,5-dibromothiophene (2.45 g,10 mmol) and Ni(dppp)Cl_2_ (0.03 g 0.55 mol%) at room temperature. The mixture was then refluxed for 20 h, then poured into a mixture of crushed ice (200 g) and 2M HCl (30 mL). The crude product was extracted with CH_2_Cl_2_ (6×50 mL). The organic phase was washed with saturated NaHCO_3_ and water, and further dried over anhydrous Na_2_SO_4_. The crude product was purified by flash chromatography (n-hexane). The pure compound was obtained as a yellow oil (2.4 g, 50.7%).^1^H NMR (500 MHz, Methylene Chloride-*d*_2_): δ 7.16 (d, *J* = 5.2 Hz, 2H), 7.04 (s, 2H), 6.93 (d, *J* = 5.2 Hz, 2H), 2.75 (t, *J* = 7.9 Hz, 4H), 1.63 (p, *J* = 7.6 Hz, 4H), 1.41-1.14 (m, 20H), 0.84 (t, *J* = 6.8 Hz, 6H).^13^C NMR (101 MHz, Methylene Chloride-*d*_2_) δ 141.18, 137.33, 131.53, 131.46, 127.32, 125.04, 33.19, 32.05, 30.85, 30.73, 30.59, 30.54, 23.98, 15.18. HR-MS (ESI, m/z): calcd for 472.2287, found m/z =472.2304.

**Figure S12**. ^1^H-NMR spectrum of **TTH_8C_**.

**Figure S13**. ^13^C-NMR spectrum of **TTH_8C_**.

# Preparation of electrodes

**Preparation of pTH electrodes.** pTH electrodes were prepared by electro-polymerization according to our previous works with some modifications.**^6^** 0.075 g of 2,2':5',2''-terthiophene (10 mM) was dissolved in 30 mL acetonitrile containing NaClO_4_ (0.366 g, 0.1 M); the solution was degassed by bubbling Ar for 10 min before polymerization. the electro-polymerization was in a three-electrode system with a saturated Ag/AgCl as the reference electrode, a graphite piece as the counter electrode and a CP (1×1 cm^2^) as the working electrode. The polyterthiophene film slowly grew on the surface of the CP electrode during the cyclic voltammetry (CV) processes, in which 15 cycles were operated in a range of 0.0 V to 1.20 V vs. *E_Ag/AgCl_* with a scan rate of 30 mV sec^−1^. The prepared electrodes were rinsed with acetonitrile and deionized water sequentially before being dried under nitrogen, labeled as pTH. The optimization process is described in Supporting Information.

**Preparation of PTTH electrodes.** Aliphatic chains were introduced on the surface of the pTH semiconductor. 0.071 g of TTH_8C_ (5 mM) was dissolved in the 30 mL electroplate liquid (CH_3_CN: DCM=9:1) containing NaClO_4_ (0.1 M). 3 cycles of CV were operated in a range of 0.0 V to 1.15 V vs. *E_Ag/AgCl_* with a scan rate of 30 mV sec^−1^. The resulting PTTH electrodes were rinsed with acetonitrile and deionized water sequentially before being dried under nitrogen.

**Preparation of catalyst-decorated photocathodes.** The catalyst-decorated electrodes were prepared according to the reported method.**^1, 7^** Rh (8.26 mg) or Rh-vi^2+^ (17.75 mg) was dissolved in 10 mL MeOH. 250 μL of the solution was cautiously transferred to the surface of PTTH electrodes by drop coating divided into 5 times. After washing with DI water and drying at room temperature, the catalyst-decorated electrodes were prepared and labeled as Rh@PTTH and Rh-vi^2+^@PTTH.

**Preparation of CoPi@BiVO_4_ photoanodes.** BiVO_4_ was prepared according to the literature.**^8, 9^** In a three-electrode cell, 0.1 M potassium phosphate solution (pH 7) containing 0.5 mM Co(NO_3_)·6H_2_O was used as precursor electrolyte, a saturated Ag/AgCl as reference electrode, and a Pt foil as counter electrode. Under simulated solar illumination (100 mW cm^−2^, incident light from the back of FTO), a bias of −0.65 V vs. Ag/AgCl was applied for 30 s.

# Supplementary Notes

## Note S1: Optimization of Polymerization for TTh and TTH_8C_ Monomers

First, the influence of different polymerization cycles of the poly-2,2':5',2''-terthiophene (pTH) photocathode was investigated (*Figure S14a*). As displayed in *Figure S15*, according to the chopping cathode current of different *x*-pTH electrodes (*x* represents the number of CV cycle for TTh). When the number of CV cycles was set as 15, the pTH exhibited a considerable photocurrent and a suitable thickness. Therefore, the 15-cycle polymerization was applied in all following experiments unless special instruction (see *Figure S16*, the scanning electron microscopy image of the pTH film displayed a nanoflower-like three-dimensional structure; see *Figure S17*, the transmission electron microscopy and EDS analysis of the pTH film).

Then, the morphology of ***y*-PTTH** films (***y*** represents the number of CV cycle for **TTH_8C_**) under different electro-polymerization conditions were observed via scanning electron microscopy. As shown in ***Figure S18***, **t**he plan-view scanning electron microscopic images of **pTH** and **y-PTTH** illustrated that when CV polymerization of **TTH_8C_** was performed at one cycle or three cycles, the morphology of **y-PTTH** slightly changed to be more stereoscopic compared with **pTH** film, however, as the number of CV polymerization cycles increased, the 3D nanoflowers on the surface turned to be slightly blunt (5 cycles), until the surface changed to be aggregated and closed into clusters (8 cycles). The gradual transformation in surface morphology confirmed the electro-polymerization growth of poly **TTH_8C_** layer. It is well known that the flower-like three-dimensional morphology is conducive to the exposure of active sites, substrate diffusion, and reduced series resistance.**^10^** Attentively, excessive TTH_8C_ polymerization could lead to the destruction of the flower-like 3D nanostructure, which is an adverse effect. Therefore, it is critical to choose a moderate condition in the polymerization procedure. Meanwhile, the water contact angle measurements displayed that **pTH** was initially hydrophobic (146.78°). The hydrophobicity of ***y*-PTTH** surface was positively correlated with the number of polymerization cycles of **TTH_8C_**, such as 151.11° for y=3 and 165.03° for y=8 (***Figure S19***). The LSV curves in PEC tests of ***y*-PTTH** indicated that immoderate **TTH_8C_** polymerization could cause its surface to be superhydrophobic, which suppressed the photocurrent and restricted liquid phase catalytic reactions (***Figure S20***). Considering these factors, an appropriate 3-cycle polymerization was adopted to fabricate alkyl chains-functionalized **PTTH** electrodes for all subsequent PEC NADH regeneration after weighing the hydrophobic self-assembled capacity and PEC performance.

Finally, the organic polymer **PTTH** electrodes were analyzedThe Raman spectra of polythiophene-based photocathodes were performed at 532 nm laser (***Figure S21***). There were the typical peaks of **pTH**, such as C-S ring deformation (697 cm^−1^), C_β_-H bending (1043 cm^−1^), C_α_ inter-ring stretching (1215 cm^−1^), and C=C ring stretching (1455 and 1495 cm^−1^).**^11^** The Fourier transform infrared spectroscopy (FT-IR) spectrum of **PTTH** is shown in ***Figure S22***. Two peaks at 2852 and 2924 cm^−1^ are observed for **PTTH**, which are attributed to the C-H bond symmetrical stretching vibration and asymmetric stretching vibration of methylene for the aliphatic chains, respectively.**^12^** This was evidence of the successful polymerization of the **TTH_8C_** monomer. Whereafter, the specific surface area was estimated by the Brunauer-Emmett-Teller (BET) experiment. As shown in ***Figure S23a****,****b***, **pTH** and **PTTH** possess similar typical H_3_-type hysteresis loops, suggesting the presence of flat slit structures,**^13^** which is consistent with the information of nanosheet-like morphology observed in SEM. Especially, the corresponding BET surface areas of **pTH** and **PTTH** were determined to be 7.89 m^2^ g^−1^ and 10.42 m^2^ g^−1^, indicating that the introduction of poly **TTH_8C_** slightly increased the specific surface area of the original polythiophene semiconductor.

## Note S2: Determination of Semiconductor Energy Band Structure

**Band gap measurement.** The light-absorption capability of home-prepared polythiophene-based organic polymer semiconductor electrodes was measured by ultraviolet−visible diffuse reflectance spectroscopy, and the corresponding band gap (***E_g_***) was calculated by ***Eq. S1***.

$(ahv)^{2}=A(hv-E_{g})^{1/2}$ ***Eq.* *S1***

Where ***a*** is the absorption coefficient, ***h*** is the Planck constant, ***ν*** is the frequency, and ***A*** is the constant.

**Ultraviolet photoelectron spectroscopy analysis.** For confirmation of the band positions, UPS plots were tested by ESCALAB Xi^+^ was employed with a photon energy of 21.2 eV. A bias (−10 V) was applied to the sample for electrons to overcome the work function of the instrument. According to the custom, the value of ***hv*** is 21.2 eV, the ***E_cutoff_*** is secondary electron cut-off edge energy, the ***E_fermi_*** is Fermi level energy, ***E_WF_*** is the work function, ***E_VB_^F^*** is the energy from the top of the valence band to Fermi level. The valence band (***VB***) and conduction band (***CB***) could be calculated according to the ***Eqs. S2***. The conversion of vacuum energy level (***E_vac_***) to standard hydrogen electrode (***E_NHE_***) is given as follows.

$$E_{WF}=hv-(E_{cutoff}-E_{fermi})$$

$$\left|E_{VB}\right|=E_{WF}+E_{VB}^{F}$$

$$CB_{vac}=VB_{vac}-E_{g}$$

$E_{vac}=-4.5-E_{NHE}$ ***Eqs.* *S2***

## Note S3: Evaluation of Photoelectrochemical Performance

**Photoelectrochemical Measurements.** A conventional H-type cell separated by a proton exchange membrane (Nafion117) was applied to assess the PEC performance. The H-type cell was O_2_-depleted with argon for 15 min before measurements. A 150 W Xenon lamp XES-40S3 simulator (SAN-EI ELECTRIC, AAA) equipped with a 420 nm cut-off filter was employed to provide simulated solar illumination. The photo-energy density was controlled at 100 mW cm^−2^ by an optical power meter (PM 100D, Thorlabs). The linear sweep voltammetry curves (LSV) were recorded with a scan rate of 20 mV sec^−1^ without iR-compensation. For tandem PEC cell measurements, in the anodic chamber, the **CoPi@BiVO_4_** photoanode in 15 mL of 0.1 M PBS (pH 7) for water oxidation; in the cathodic chamber, as-prepared photocathode was immersed in an argon-saturated 15 mL of 0.1 M PBS (pH 7) containing 0.5 mM NAD^+^. Two solar simulators were simultaneously employed to both photoanode (via back-side illumination) and photocathode (via front-side illumination with a 420 nm cut-off filter) without applying external bias.

**The Incident Photon to Current Efficiency (IPCE).** The IPCE measurements of the catalyst-modified polythiophene-based photocathodes were directly measured by the photoelectrochemical workstation (CIMPS-2, Zahner) at a constant applied potential of 0.1 V vs. RHE. The integral current was calculated by the ***Eq. S3*** to compare with the corresponding photocurrent in PEC measurement. (Where λ from 365 nm to 800 nm)

$Integral_{j}=\int_{\lambda} \frac{1}{1240}\times IPCE\times P_{mono}\cdot d\lambda$ ***Eq. S3***

**PEC NADH Regeneration and NADH Quantification.** Ordinarily, the formation of apparent NADH was detected by UV–vis spectrophotometry at the 340 nm absorption wavelength. UV–vis absorption peaks at 340 nm for different concentrations of commercial NADH were measured to determine the linear relation between absorbance (***A_sta.NADH_***) and concentrations of NADH (***C_sat.NADH_***), combined with the *Lambert-Beer Law*. Therefore, the concentration and yield of NADH regenerated by PEC reaction can be calculated from ***A_reg.NADH_*** according to ***Eqs. S4*** and ***S5***.

$A_{sta.NADH}=\varepsilon\times l\times c_{sta.NADH}+b$ ***Eq. S4***

$Yield(\%)=\frac{C_{reg.NADH}}{C_{\text{initial. NAD}^{\text{+}}}}\times100\%$ ***Eq. S5***

Where ***ε*** (cm^−1^ M^−1^) is the molar absorption coefficient of NADH, ***l*** (1 cm) is the optical distance of quartz cuvette, ***C_sta.NADH_*** (M^−1^) is the concentration of standard NADH, and ***b*** is the constant.

**Faradaic Efficiency Determination.** The Faradaic efficiency of NADH regeneration reaction through the PEC approach can be obtained by ***Eq. S6*** Where ***n*** is the number of electrons transferred in NADH regeneration reactions (n=2), ***F*** is the Faraday constant (96485 C mol^−1^), ***N_real_*** (mol) is the NADH molarity, ***V*** is electrolyte volume (V=15 cm^3^), ***Q_i-t_*** is the amount of charge passing through the electrode during the PEC reaction.

$FE(\%)=\frac{N_{real}}{N_{theory}}=\frac{nFVN_{real}}{Q_{i-t}}\times100\%$ ***Eq. S6***

**The Applied Bias Photon-to-Product Efficiency (ABPE)**. ABPEs of the catalyst-modified polythiophene-based photocathodes for NADH regeneration were given according to the ***Eq. S7*** by converting LSV curves of the PEC test and faradaic efficiency of NADH at a given bias.

$\text{A}BPE_{NADH}(\%)=\frac{(V_{bias}-V_{redox})\times\left|J_{light}-J_{dark}\right|}{P_{light}}\times\eta_{NADH}$ ***Eq. S7***

Where ***V_bias_*** is the applied potential and ***V_redox_*** is the thermodynamic potential (V verse RHE) of the NAD^+^/NADH reaction. ***P_light_*** (100 mW cm^−2^) is the optical power density of AM 1.5G and ***η_NADH_*** corresponds to the FE at a specific bias. Here, ***V_redox_*** = ***V_vs.NHE_*** (NAD^+^/NADH) + 0.0592 × pH = -0.32+0.0592 × 7 = 0.094.

**Turnover Frequency (TOF) Calculation.** The TOFs of two kinds of Rh-catalyst assembled on polythiophene-based photocathodes were determined according to ***Eq. S8***, where ***C_NADH_*** is the concentration of NADH (mM) obtained through the PEC reaction, ***S*** (cm^2^) is the surface area in the test, ***Γ_Rhcat_*** (mol cm^−2^) is the amount of Rh(III) active sites of the assembled catalyst on the electrode and ***T*** is the reaction time (h).

$TOF=\frac{C_{NADH}}{S\times\tau_{Rhcat}\times T}$ ***Eq. S8***

**PEC-Enzyme Coupled Synthesis of Glutamate.** The conversation of α-ketoglutarate to glutamate was achieved by adding the substrate including α-ketoglutarate (0.5 mM), (NH_4_)_2_SO_4_ (0.1 M), and GDH (50 U mL^-1^) to the NADH-containing electrolyte generated by PEC reaction. The mixture was maintained for 2 hours to have a sufficient enzymatic reaction. Given the high selectivity of GDH, the glutamate production was determined from the conversion of α-ketoglutarate by HPLC equipped with a C_18_ column. (Eluent: 0.1 vol % phosphoric acid, flow rate: 1.0 mL min^−1^, detect wavelength: 214 nm)

## Note S4: Investigation of Charge Transport Dynamics

**Photoelectrochemical Impedance Spectroscopy (PEIS).** PEIS measurements of catalyst-modified polythiophene-based photocathodes were performed by applying 100K to 0.1 Hz frequency range with an AC voltage amplitude (5 mV) in a 0.1 M phosphate buffer (PBS, pH 7) at a bias of 0.2 V vs. RHE under solar-simulator illumination (100 mW cm^−2^).

**Film Resistivity Test by Four-point Probe Method**. The film resistivity (***ρ***) of Rh complex-modified polythiophene-based photocathodes was determined by a Hall 8800. According to the ***Eq. S9***, ***δ*** is the thickness of the electrode and *R* is the resistance. ***k*** is the instrument parameter. The thickness of two kinds of Rh complex complex-modified layer was detected to be 233±3 μm and 235±3 μm for by stylus profilometer, respectively.

$\rho=kR\delta$ ***Eq. S9***

**Electronic Property by Atomic Force Microscope.** The AFM height sensor topography image of films was applied to display microstructure with a scan size of 1×1 μm^2^ area. The conductive atomic force microscope model (C-AFM) was performed to assess the electrical properties of the films resulting in high-resolution current distribution 2D images with a scan size of 1×1.5 μm^2^ area at a scan rate of 1.0 Hz.

**Photoluminescence (PL) Spectroscopy** **Analysis.** Steady-state PL emission spectra were monitored at 450 nm of excitation wavelength with a 5 nm of slit width. Time-resolved PL emission decay spectra were recorded under a laser of 450 nm wavelength and were fitted by the biexponential function (***Eq.* *S10***); The average fluorescence lifetimes were calculated by ***Eq.* *S11***.

$I(t)=A+K_{1}\exp^{-t/\tau_{1}}+K_{2}\exp^{-t/\tau_{2}}$ ***Eq.* *S10***

*$\tau_{\text{average}}\text{ =}\frac{K_{1}\tau_{1}^{2}+K_{2}\tau_{2}^{2}}{K_{1}\tau_{1}+K_{2}\tau_{2}}$* ***Eq.* *S11***

Where *K*_1_ and *K*_2_ are the correlative amplitudes, and ***τ*_1_** and ***τ*_2_** are the slow and lifetime constant, respectively. ***τ*_average_** represents the average fluorescent lifetime.

# Supplementary Figures


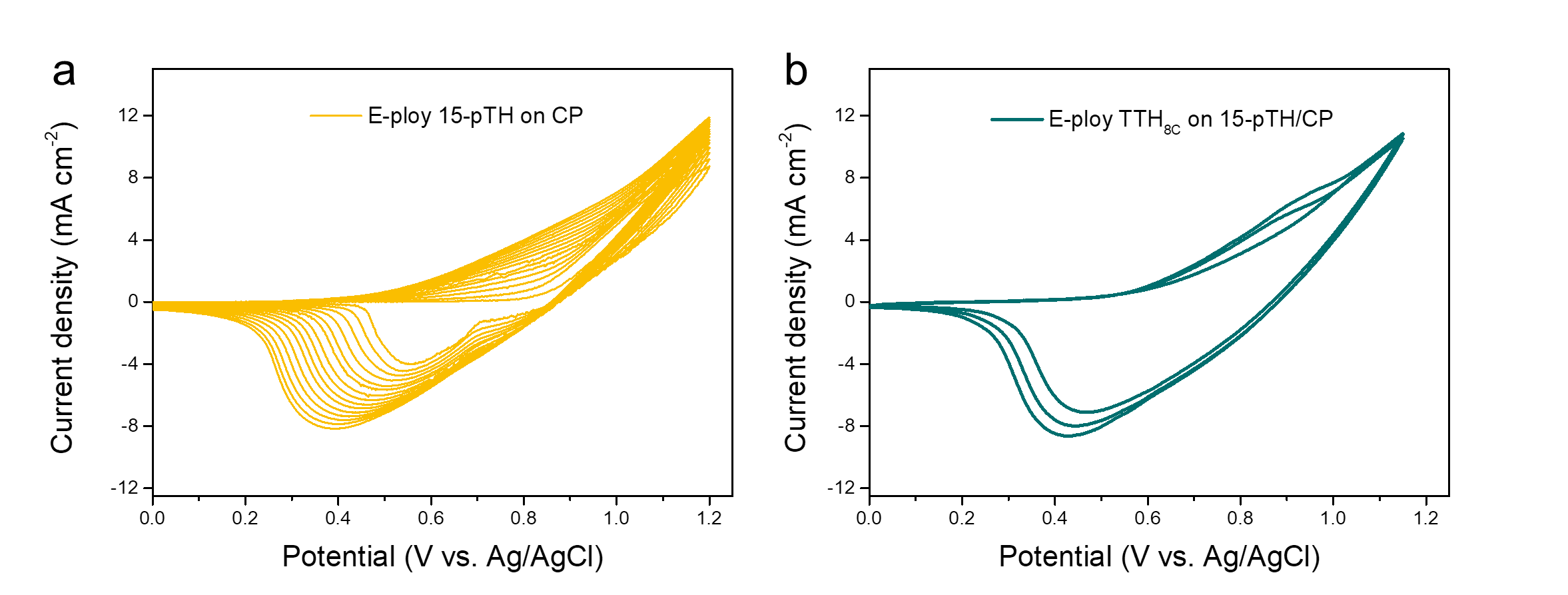


**Figure S14**. Electro-polymerization cyclic voltammetry curves for the preparation of (a) **pTH** and (b) **PTTH**. (Only the case of x=15, y=3 was displayed)

**Figure S15**. Chopped illumination I-V curves of **x-pTH** electrodes tested in 0.1 M PBS 7 + 0.5 mM NAD^+^ with a scan rate of 20 mV s^−1^. (x=5, 15, 25)


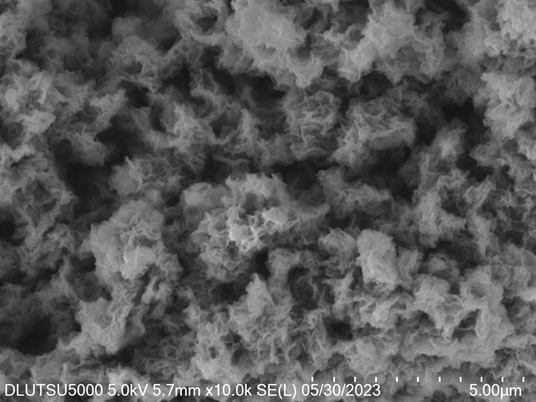


**Figure S16**. SEM image of pure **pTH** by electro-polymerization.


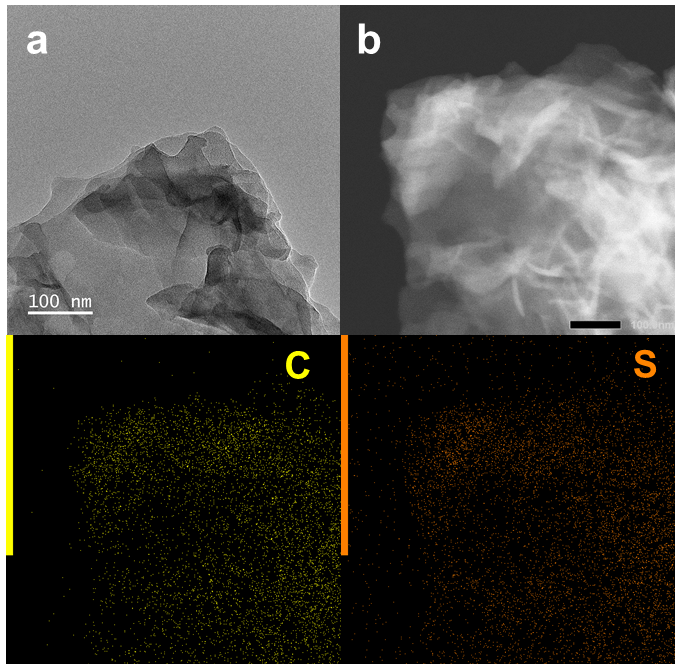


**Figure S17**. (a) High-resolution transmission electron microscopy (TEM) image of the **pTH** film (b) HAADF-STEM image with EDS analysis of the **pTH** film. Elements detected: C and S, which were evenly dispersed in the nanosheets of the **pTH**.


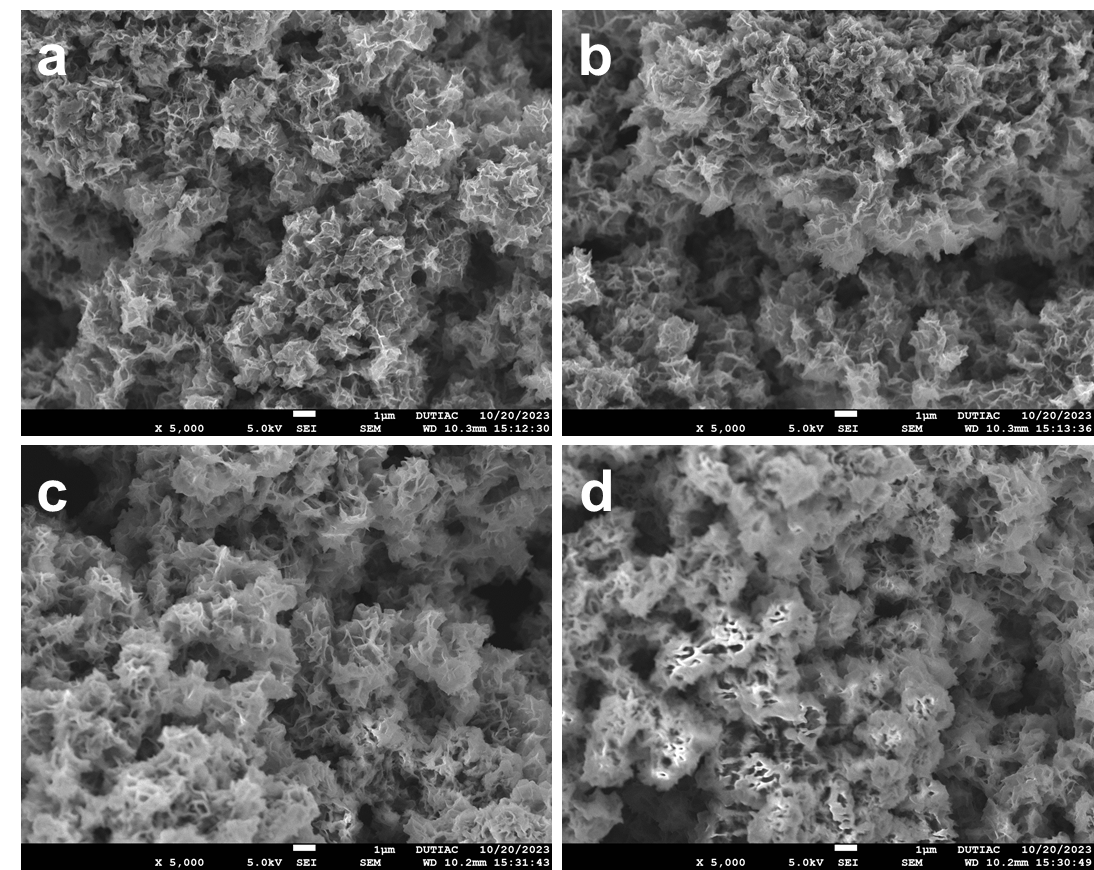


**Figure S18**. SEM images of (a) **1-PTTH**, (b) **3-PTTH**, (c) **5-PTTH**, and (d) **8-PTTH**.


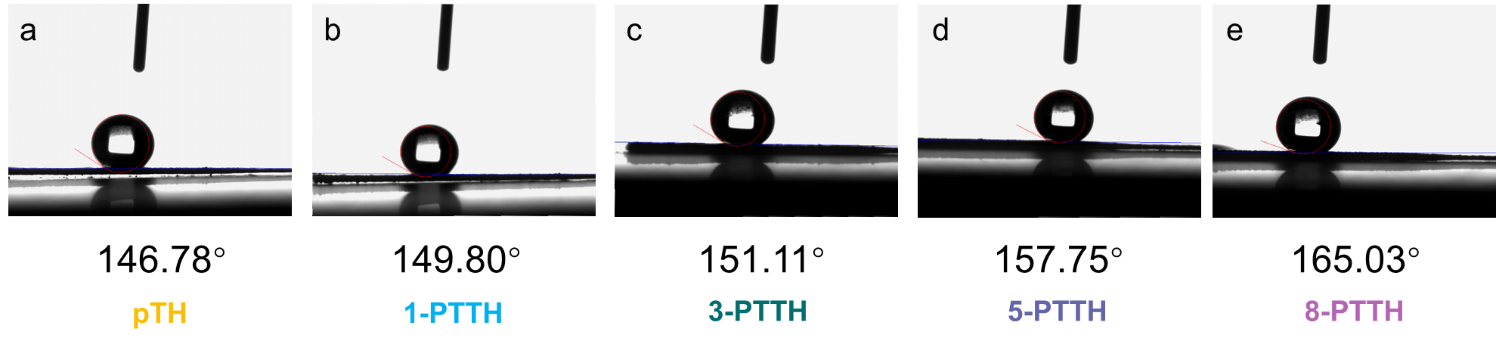


**Figure S19**. Water contact angle tests of (a) **pTH**, (b) **1-PTTH**, (c) **3-PTTH**, (d) **5-PTTH**, and (e) **8-PTTH**.

**Figure S20**. Chopped illumination I-V curves of **pTH**, **1-PTTH**, **3-PTTH**, **5-PTTH**, and **8-PTTH** electrodes tested in 0.1 M PBS 7 + 0.5 mM NAD^+^ with a scan rate of 20 mV s^−1^.

**Figure S21**. Raman spectra of **y-PTTH** films on the CP substrate. (y=0,1,3,5,8)

**Figure S22**. FT-IR spectroscopy of **pTH** (yellow) and **PTTH** (cyan) films on the CP substrate.


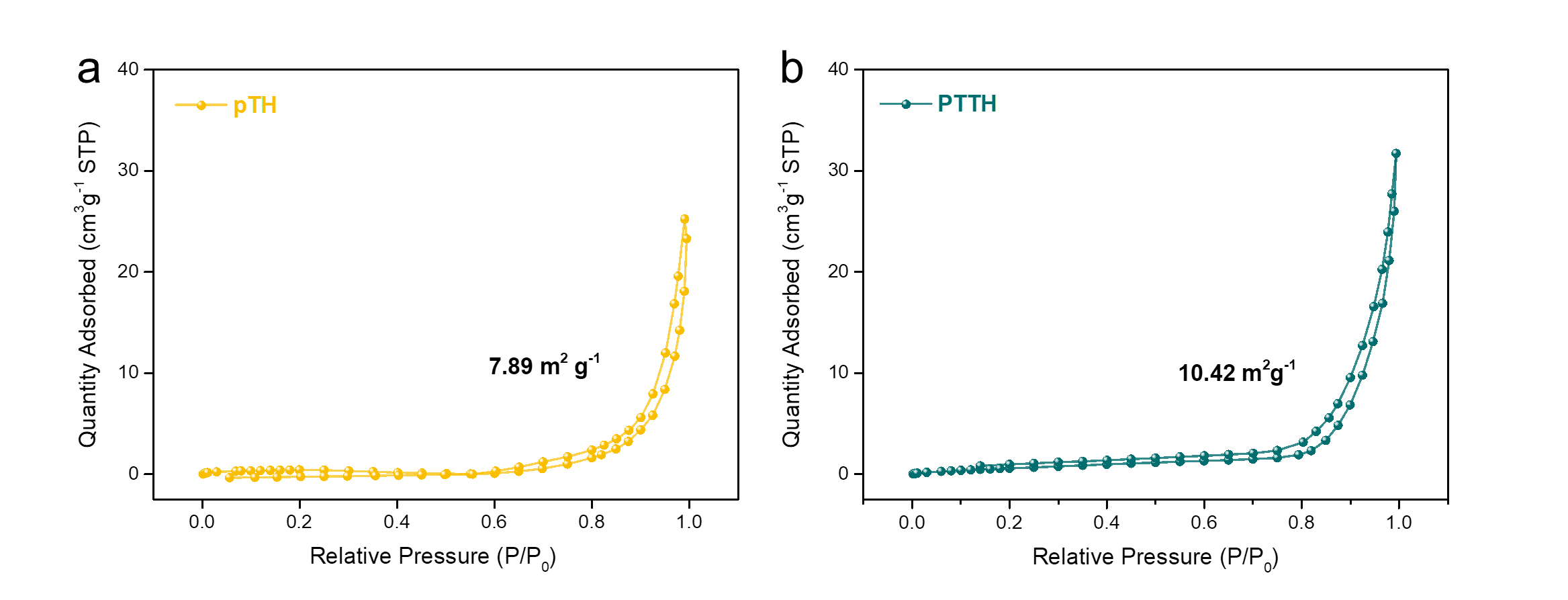


**Figure S23**. N_2_ adsorption-desorption isotherm curves of (a) **pTH** and (b) **PTTH** on the CP substrate.


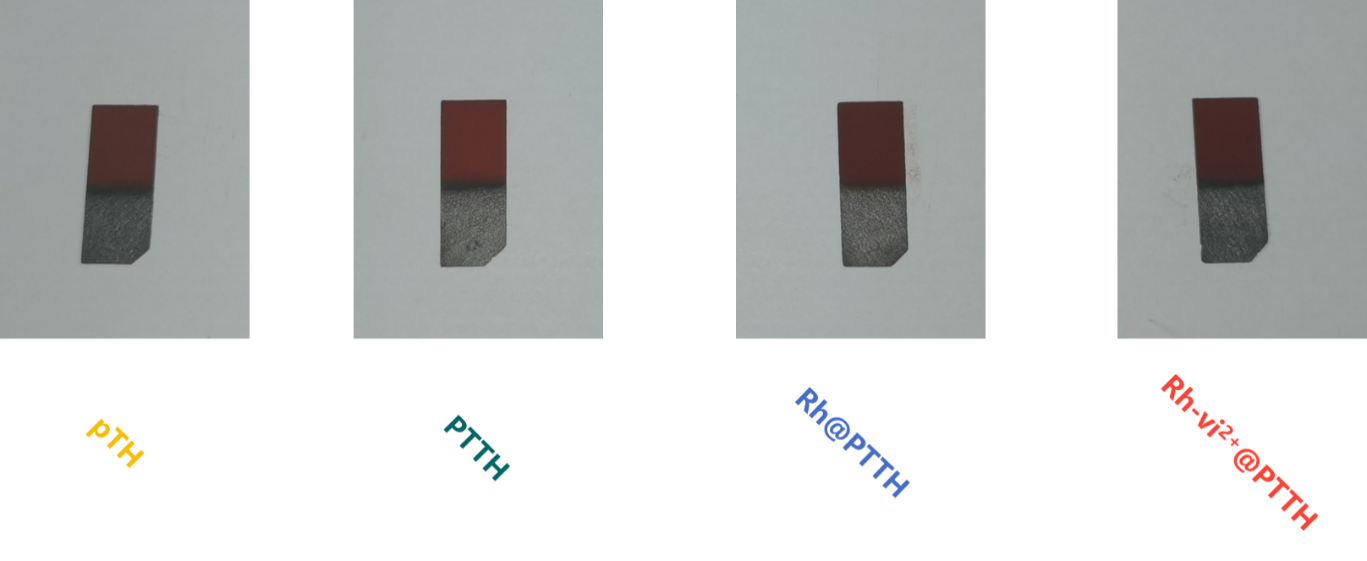


**Figure S24**. Digital photos of photocathodes: **pTH**, **PTTH**, **Rh@PTTH**, and **Rh-vi^2+^@PTTH** films on the CP substrate.

**
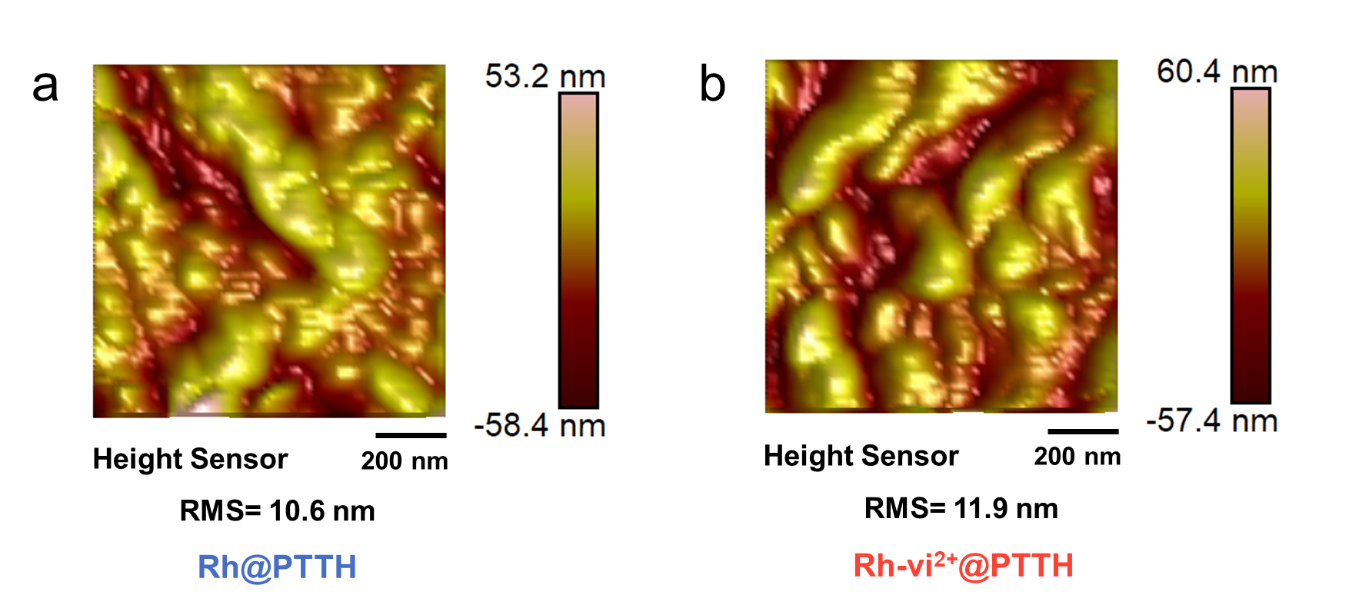
**

**Figure S25**. AFM topography images of (a) **Rh@PTTH** and (b) **Rh-vi^2+^@PTTH**. (RMS stands for surface roughness)


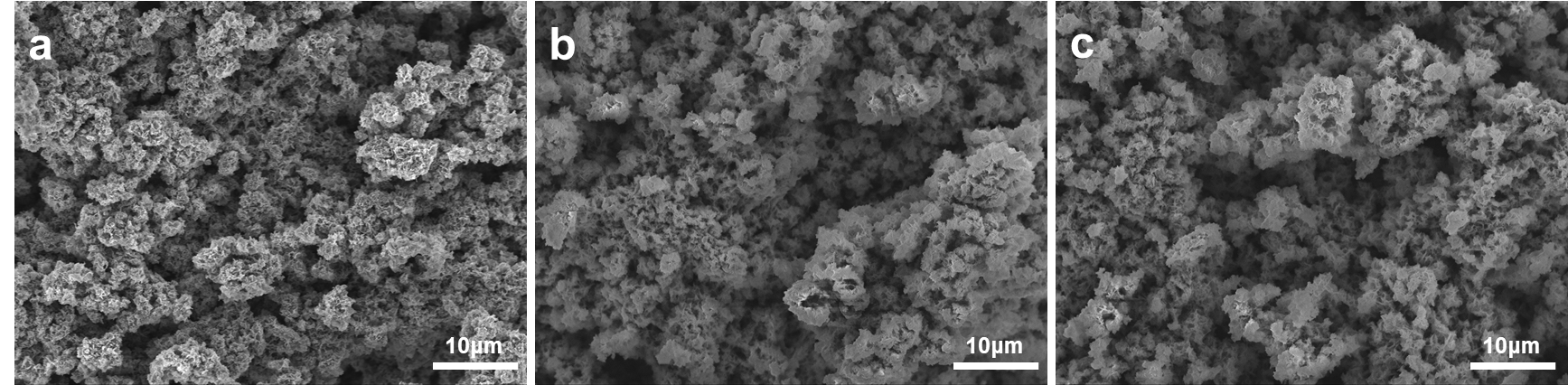


**Figure S26**. SEM images of (a) **PTTH**, (b) **Rh@PTTH**, and (c) **Rh-vi^2+^@PTTH**.

**
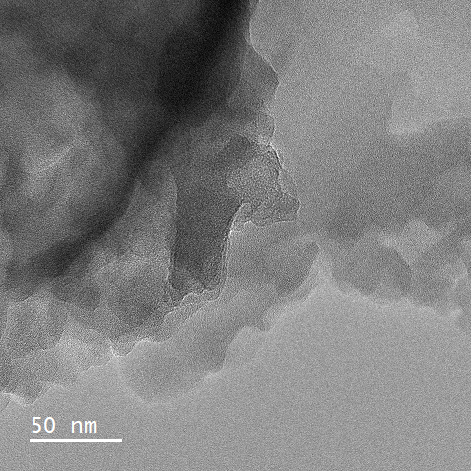
**

**Figure S27**. High-resolution transmission electron microscopy (HRTEM) image of **PTTH** film.

**
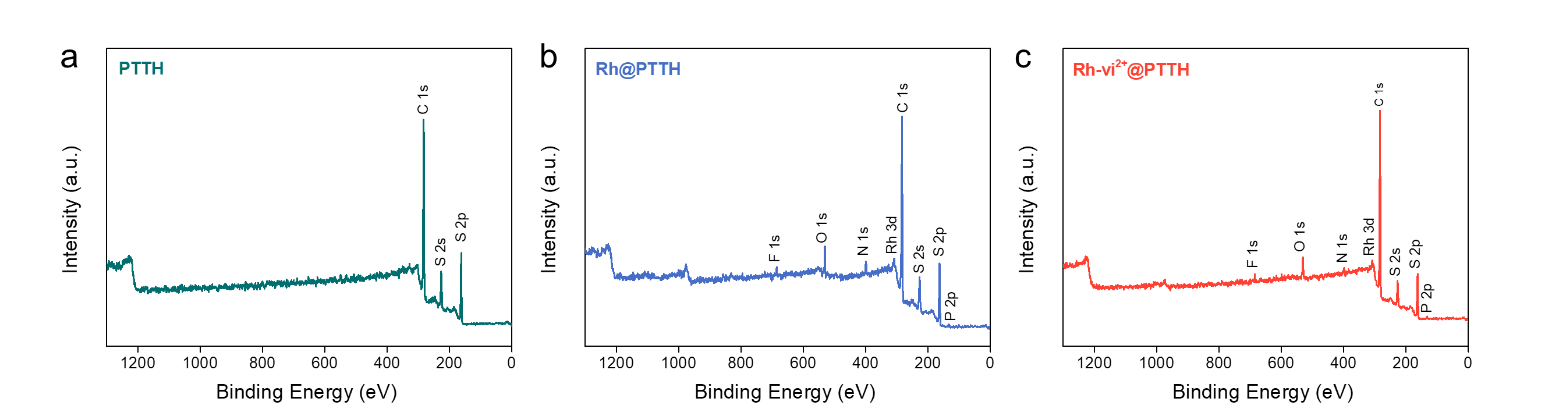
**

**Figure S28**. XPS survey spectra of (a) **PTTH** (b) **Rh@PTTH** and (c) **Rh-vi^2+^@PTTH**.

**
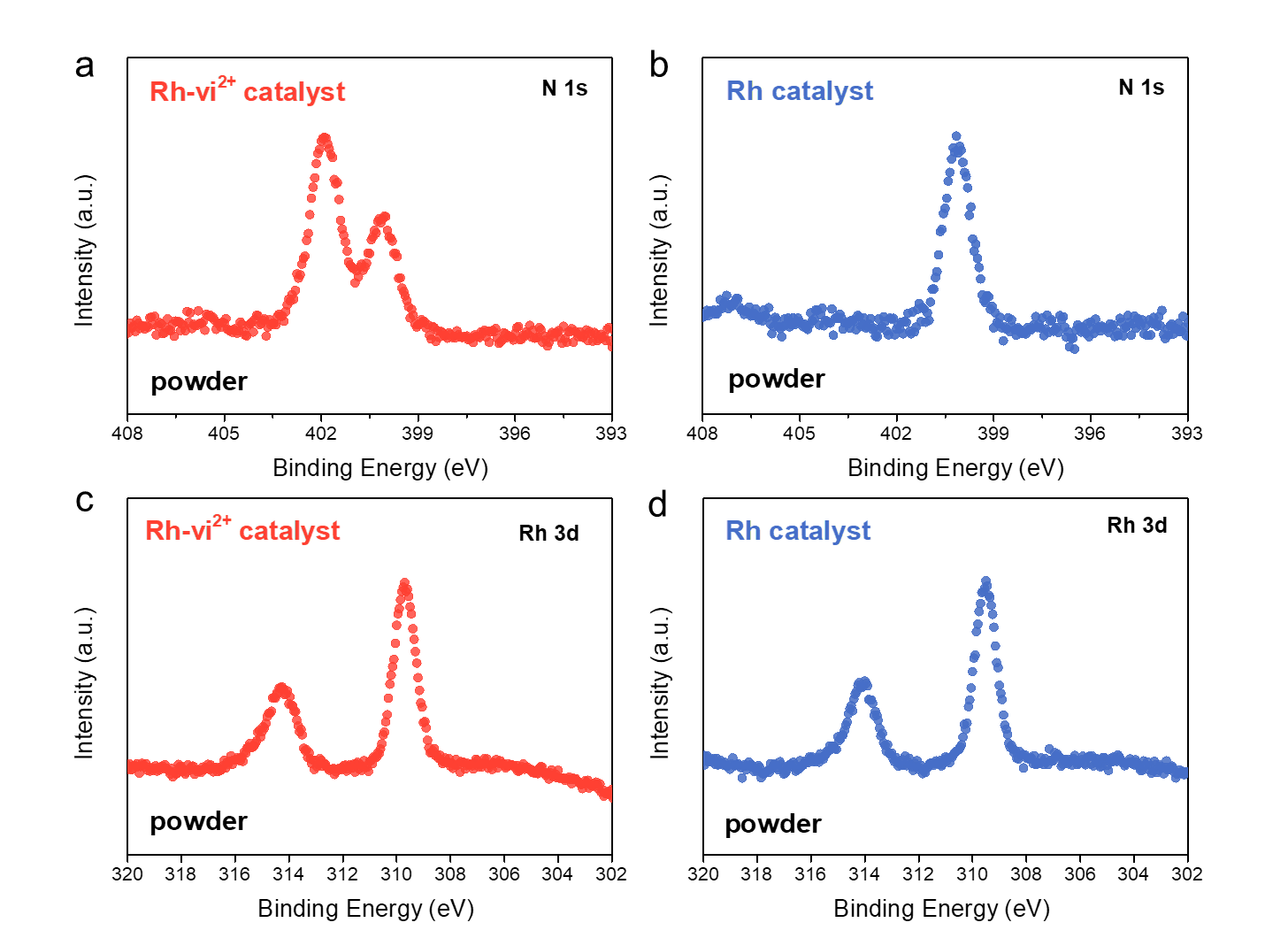
**

**Figure S29**. High-resolution XPS spectra of Rh 3d of powder catalyst **Rh** (a), **Rh-vi^2+^** (c) and N 1s of powder catalyst **Rh** (b), **Rh-vi^2+^** (d).


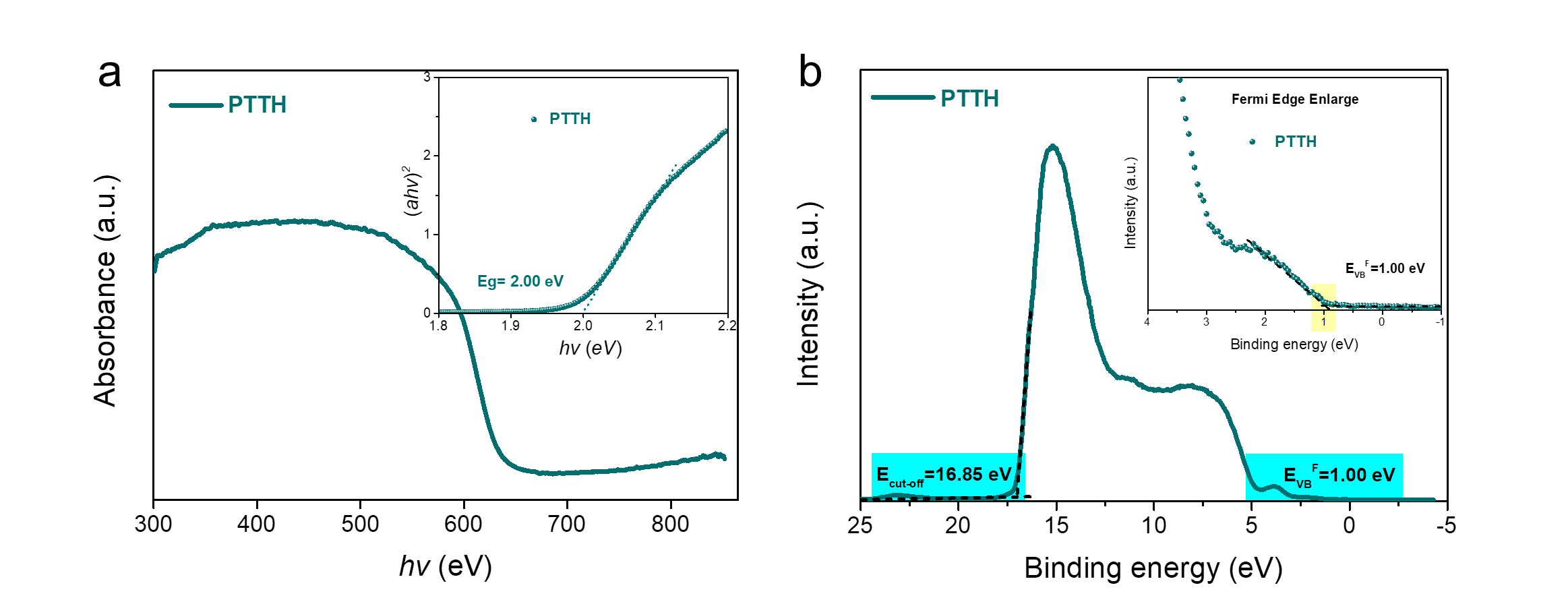


**Figure S30**. (a) UV-vis diffuse reflectance spectrum of **PTTH**. (Inset: corresponding Tauc plots, the band gap was determined to be 2.00 eV.) (b) UPS spectra of **PTTH** for VBM determination. The enlarged inset showed the corresponding valence band position.

For **PTTH** electrode:

│E*_WF_*│=21.22−(16.85−0) =4.37 eV; │E*_VB_*│=4.37+1.00=5.37 eV;

VB = −5.37 V vs. vacuum = −4.5−(−5.37) = 0.87 V vs. NHE = 1.28 V vs. RHE.

CB = VB−E_g_ = −5.37+2.00 = −3.37 vs. vacuum = −4.5−(−3.37) = −1.13 V vs. NHE = −0.72 V vs. RHE.


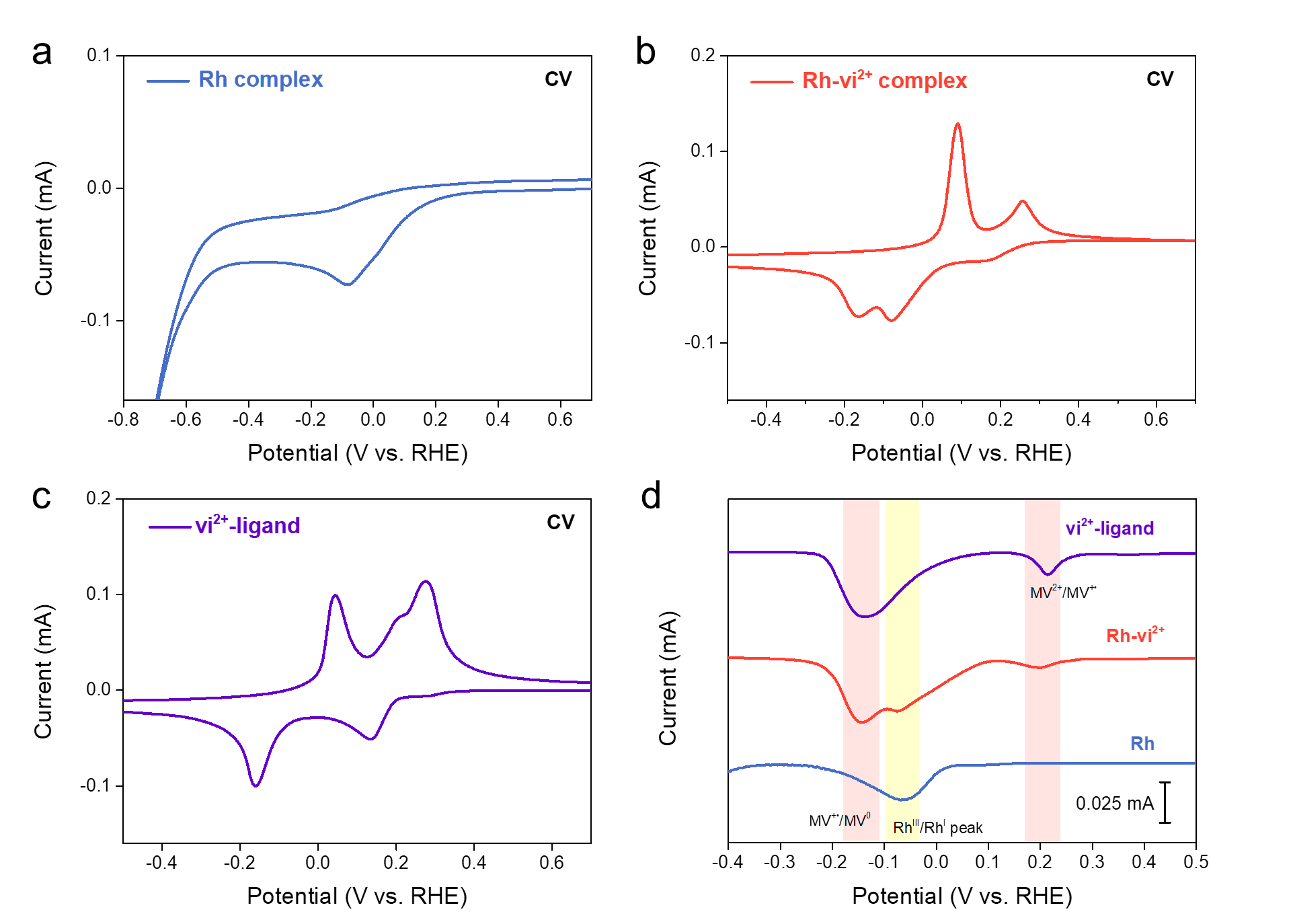


**Figure S31**. Cyclic voltammogram of (a) 0.1 mM **Rh** and (b) 0.1 mM **Rh-vi^2+^** and (c) 0.1 mM **vi^2+^-ligand** tested in 0.1 M PBS 7 aqueous solution using a glassy carbon working electrode with a scan rate of 100 mV s^−1^. (d) Differential pulse voltammetry (DPV) analysis of **Rh**, **vi^2+^-ligand** and **Rh-vi^2+^** under an amplitude of 50 mV.

**Electrochemical measurements:** On a CHI 660e electrochemical workstation, cyclic voltammetry (CV) was performed in a conventional three-electrode cell equipped with a glassy carbon disc (φ = 3 mm) as a working electrode, a saturated Ag/AgCl as the reference electrode and a platinum mesh as the counter electrode. The reference electrode was corrected to 0.195 V vs. normal hydrogen electrode (NHE). The working glassy carbon was polished before each experiment. The supporting electrolyte phosphate buffer (0.1 M PBS, pH 7, dissolving 8.46 g Na_2_HPO_4_ and 4.97 g NaH_2_PO_4_ in 1.0 L deionized water) containing 0.1 mM **vi^2+^-ligand** or 0.1 mM **Rh-vi^2+^** was degassed by purging with argon for 10 min. The scan rate of voltammograms was set as 100 mV sec^−1^; Under the same experimental conditions, the DPV curves of the **vi^2+^-ligand** and **Rh-vi^2+^** were respectively tested at the range of -0.4 V to 0.5 V vs. RHE with an amplitude of 50 mV.


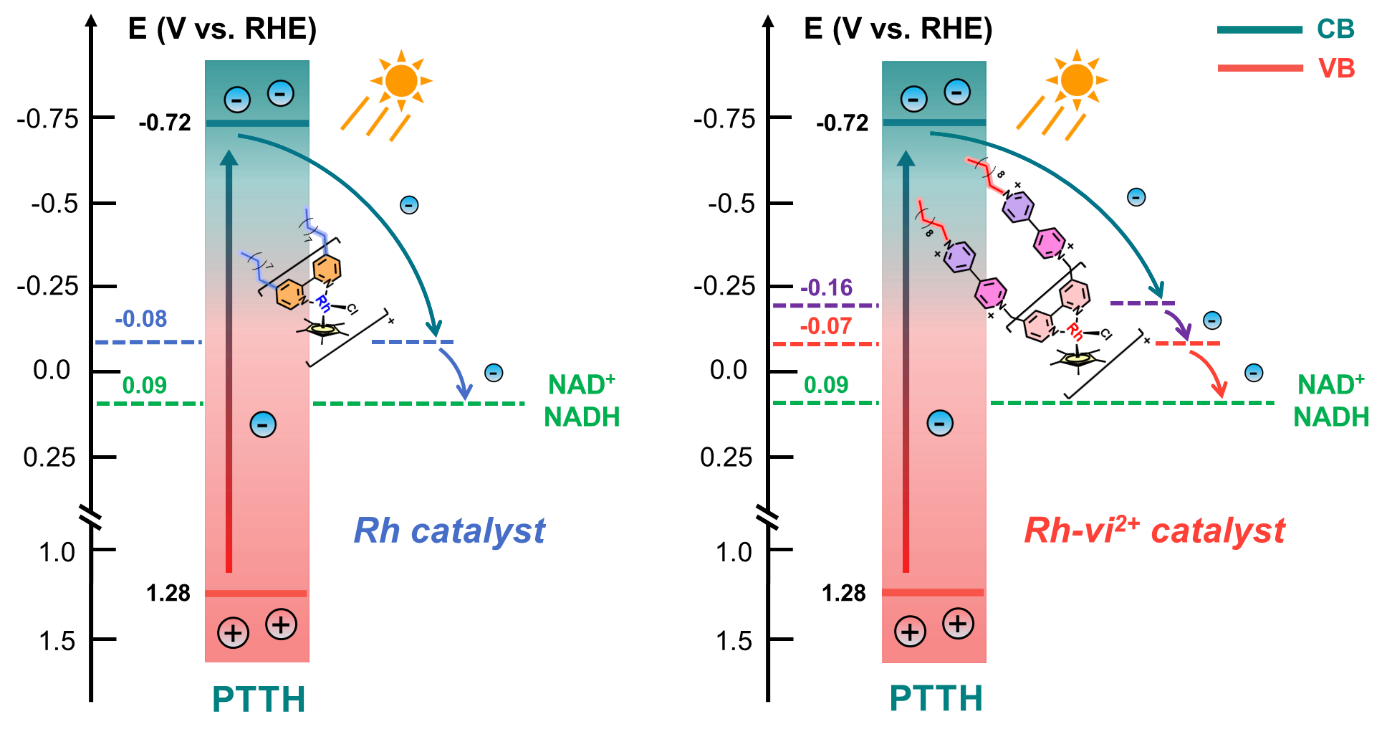


**Figure S32**. Energy-band alignment schematic diagram of **PTTH**, **Rh** (left), and **Rh-vi^2+^** (right).

**
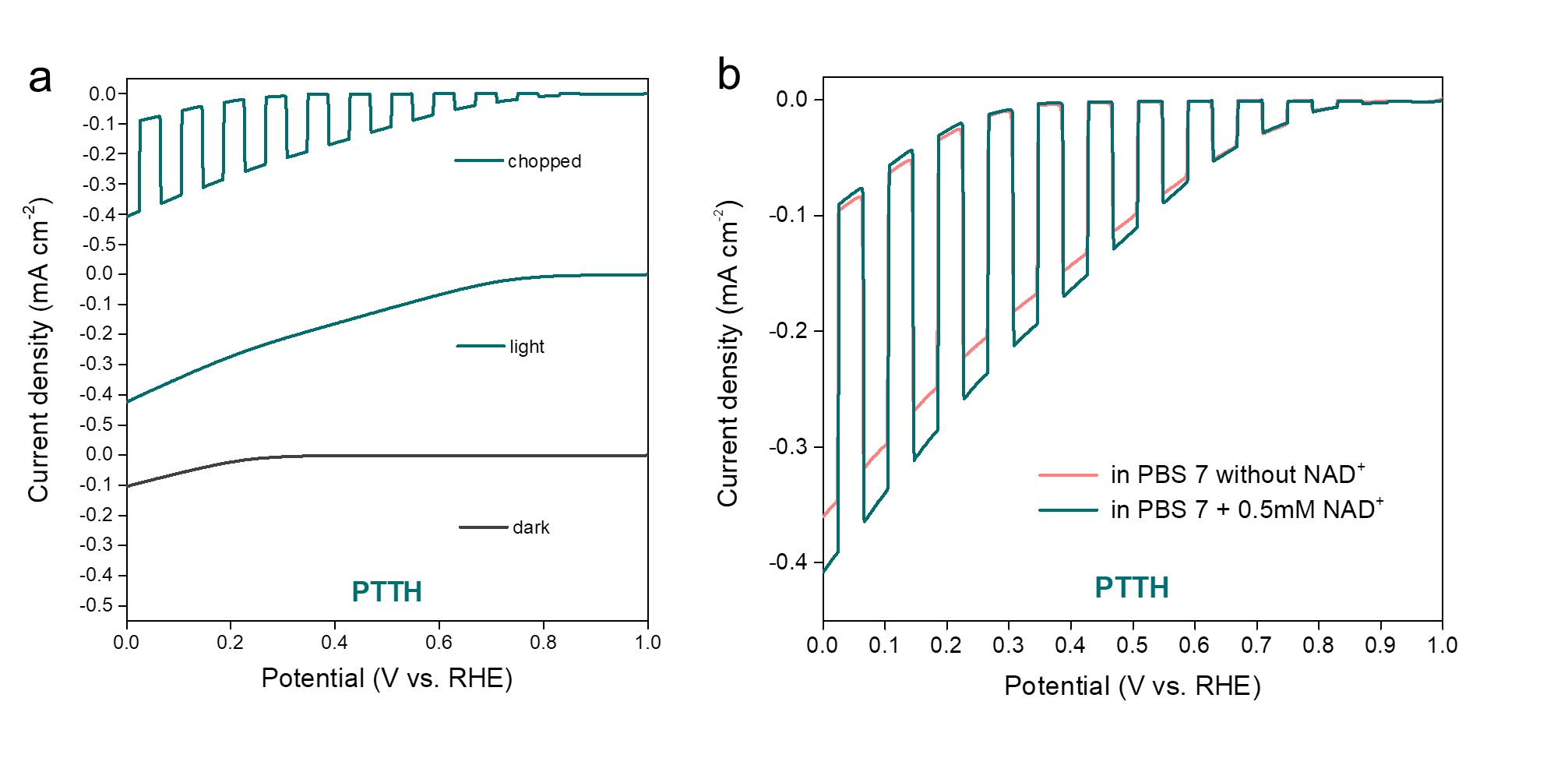
**

**Figure S33**. (a) LSV curves of **PTTH** photocathode in 0.1 M PBS 7 + 0.5 mM NAD^+^ with a scan rate of 20 mV s^−1^. (Under illumination, dark and chopped illumination conditions) (b) Comparison of the I-V curves of the **PTTH** photocathode under chopping illumination in PBS 7 with 0.5 mM NAD^+^ or not.

**
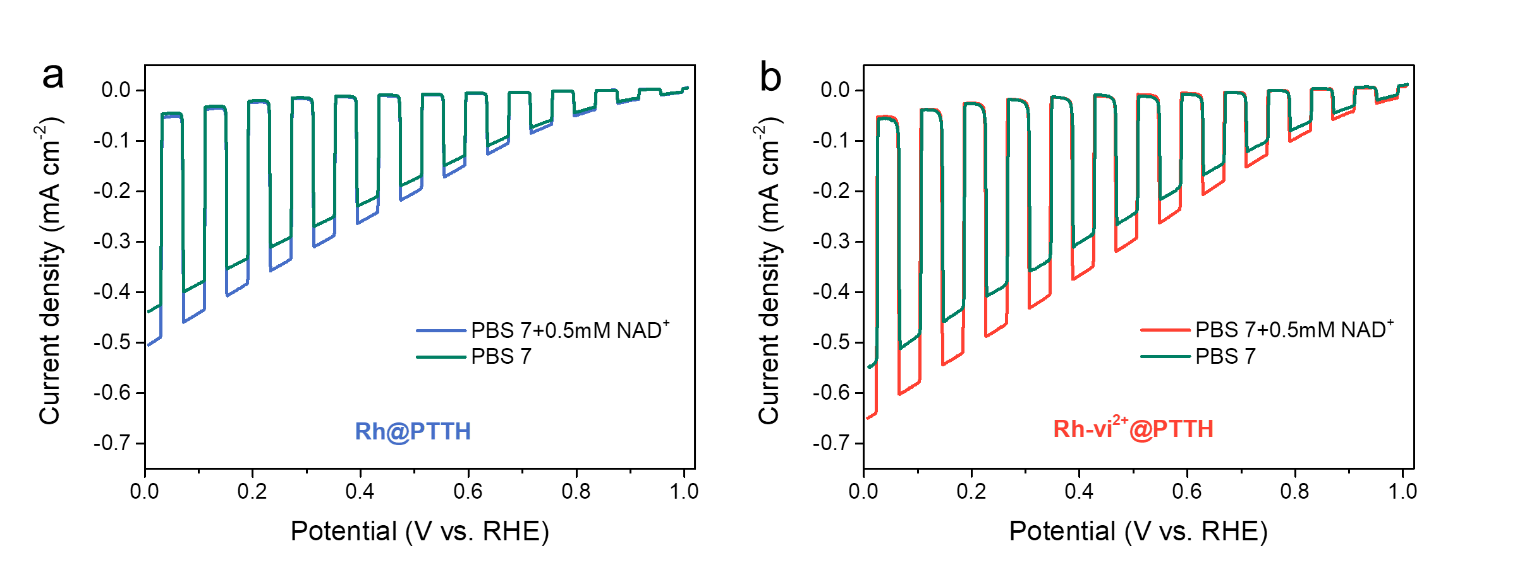
**

**Figure S34**. Chopped illumination I-V curves of (a) **Rh@PTTH** and (b) **Rh-vi^2+^@PTTH** photocathodes in PBS 7 + NAD^+^ (0 or 0.5 mM) with a scan rate of 20 mV s^−1^.

**Figure S35**. LSV curves of **Rh@PTTH** and **Rh-vi^2+^@PTTH** photocathodes in 0.1 M PBS 7 + 0.5 mM NAD^+^ under dark or light conditions.

**
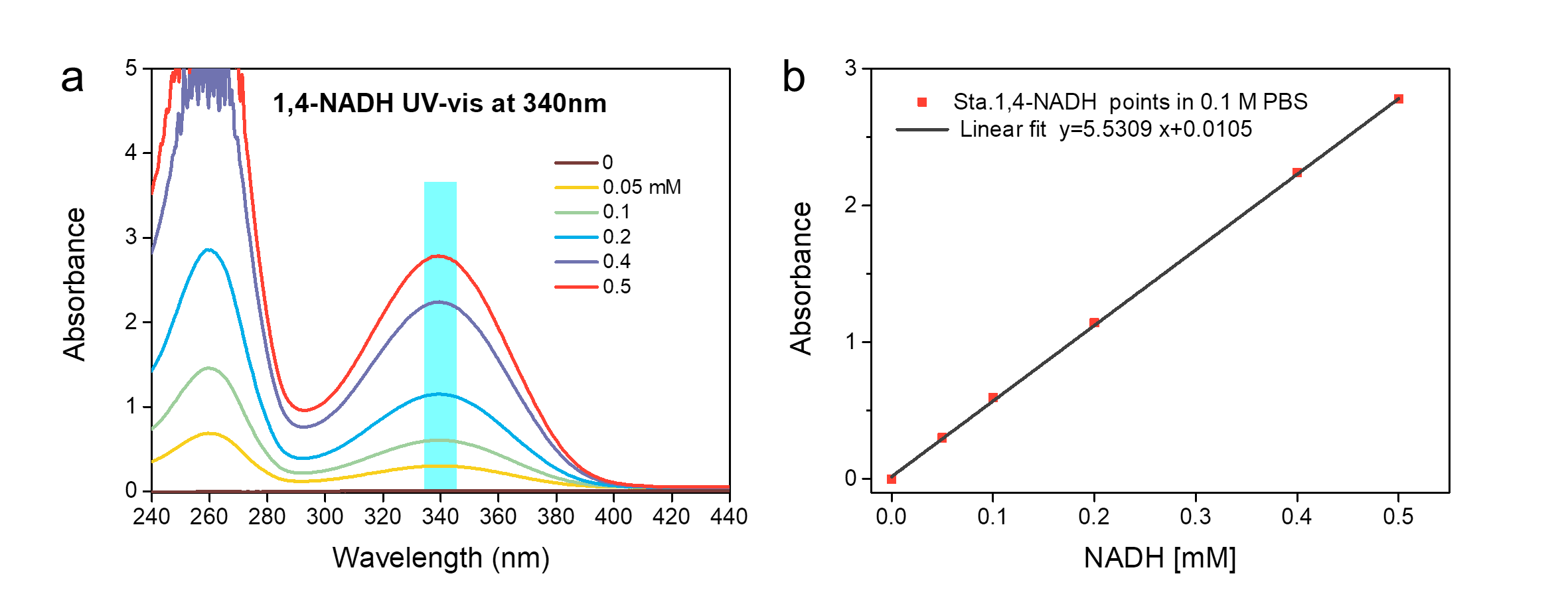
**

**Figure S36**. UV-vis absorption spectra of known concentration of commercial 1,4-NADH (0, 0.05, 0.1, 0.2, 0.4, 0.5 mM in PBS 7). (b) The corresponding calibration curve between UV-vis absorption at 340 nm and the concentration of 1,4-NADH. (y=5.5309 x+0.0105, R^2^=0.9997)

**
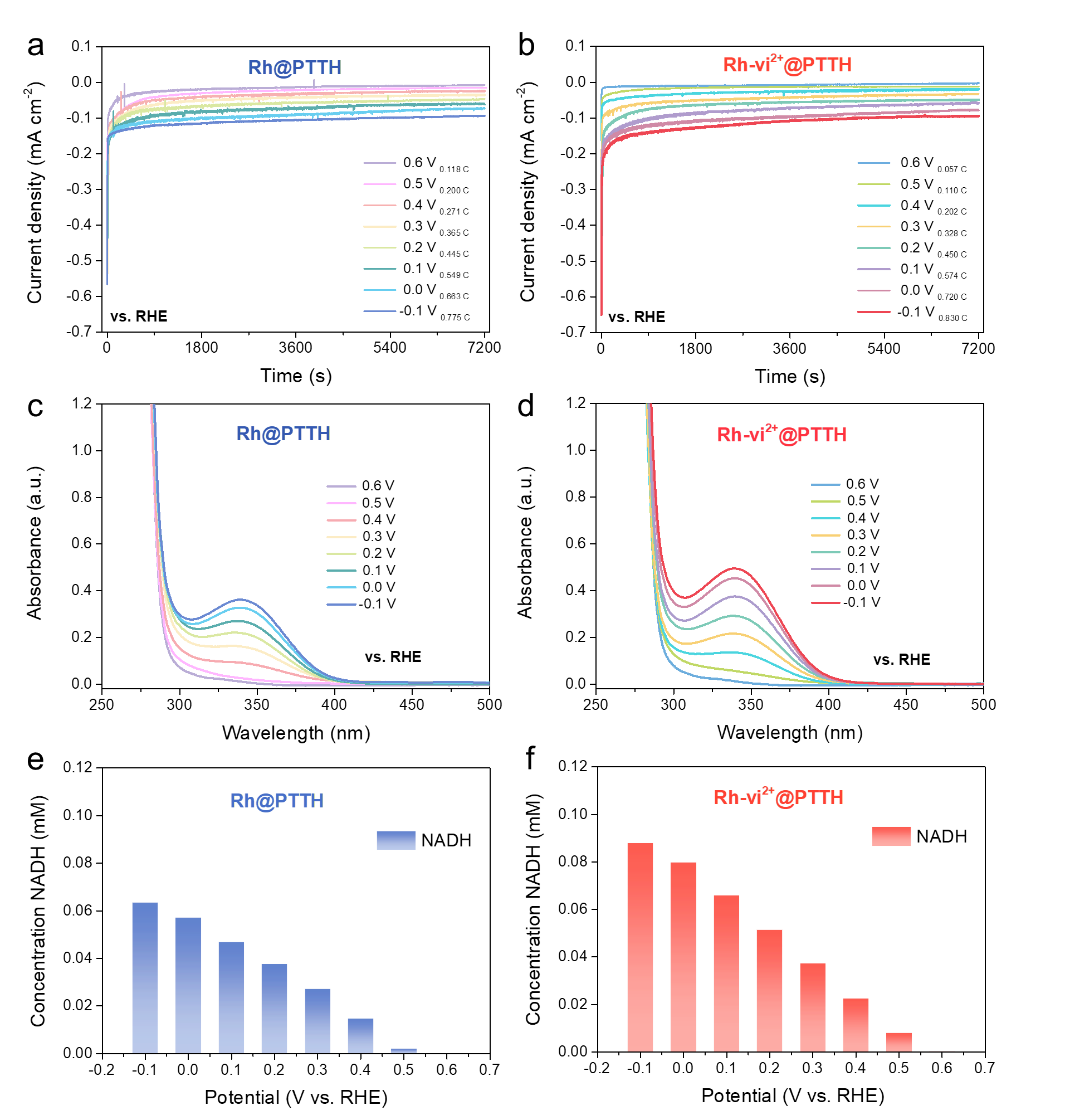
**

**Figure S37**. Chronoamperometry curves at different constant potentials of (a) **Rh@PTTH** and (b) **Rh-vi^2+^@PTTH**. The amount of charge. UV-vis absorption spectra of the electrolyte at different potentials after 2 hours of chronoamperometry electrolysis for (c) **Rh@PTTH** and (d) **Rh-vi^2+^@PTTH**. The corresponding NADH concentration (e) and (f) after 2 hours of PEC measurements.


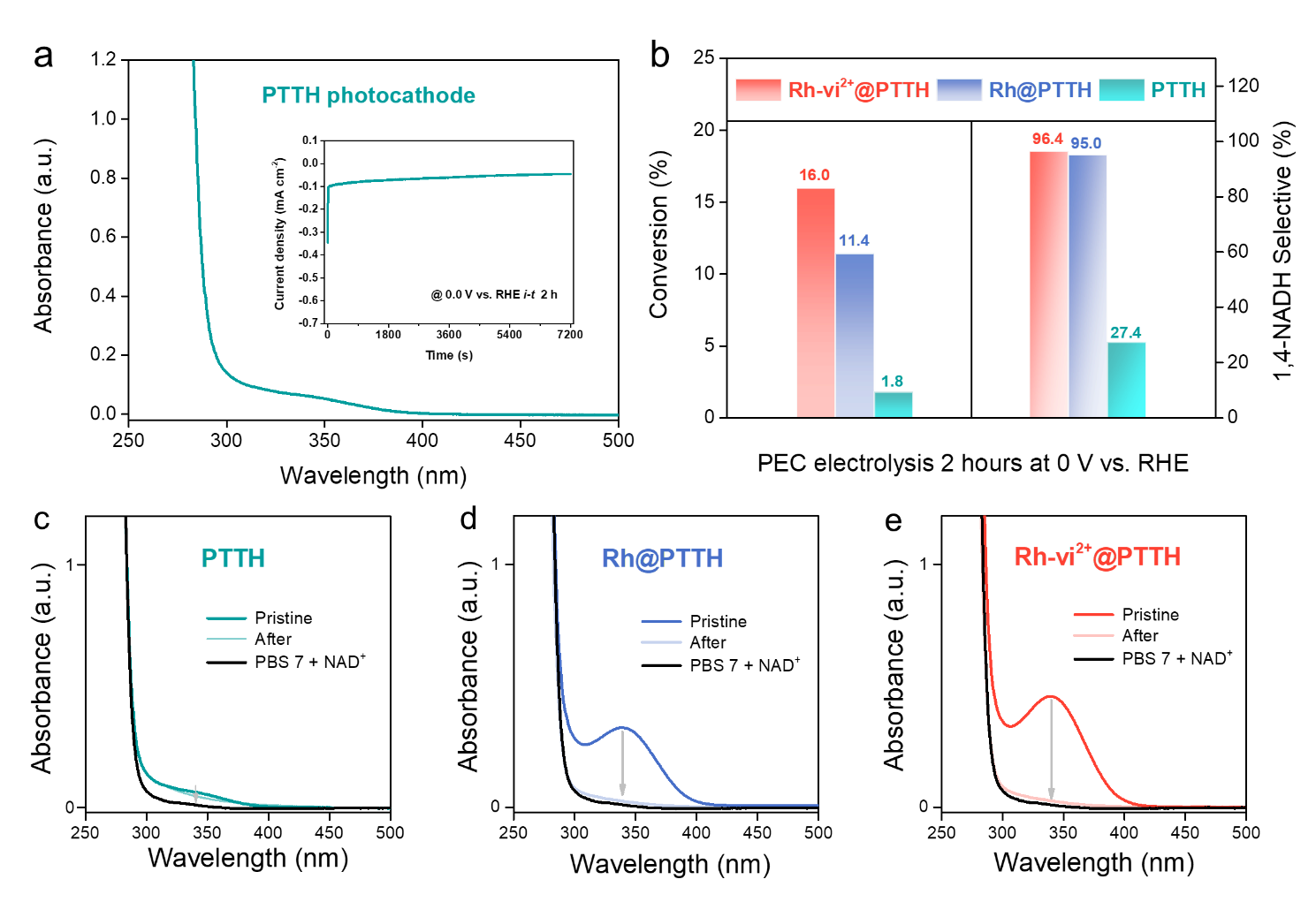


**Figure S38**. (a) UV-vis absorption spectra of the electrolyte at 0 V vs. RHE after 2 hours of chronoamperometry electrolysis for **PTTH** photoelectrode. (Inset: corresponding i-t curve) (b) Comparison of conversion rate and reduction product (1,4-NADH) selectivity at the 0.0 V vs. RHE for **PTTH**, **Rh@PTTH**, **Rh-vi^2+^@PTTH** and photocathodes. UV-vis absorption detection at 340 nm after enzymatic reaction of **PTTH** (c), **Rh@PTTH** (d), **Rh-vi^2+^@PTTH** (e).

For verifying the selectivity of the reduction product, the characteristic absorption at 340 nm was detected before (*A_initial_*) and after (*A_after_*) the enzymatic reaction, and the selectivity was obtained according to ***Eq. S12*** (*A_0_* was the absorption at 340 nm of PBS 7 + 0.5 mM NAD^+^ mixture with no reaction).^14-16^

** ***Eq. S12***

**
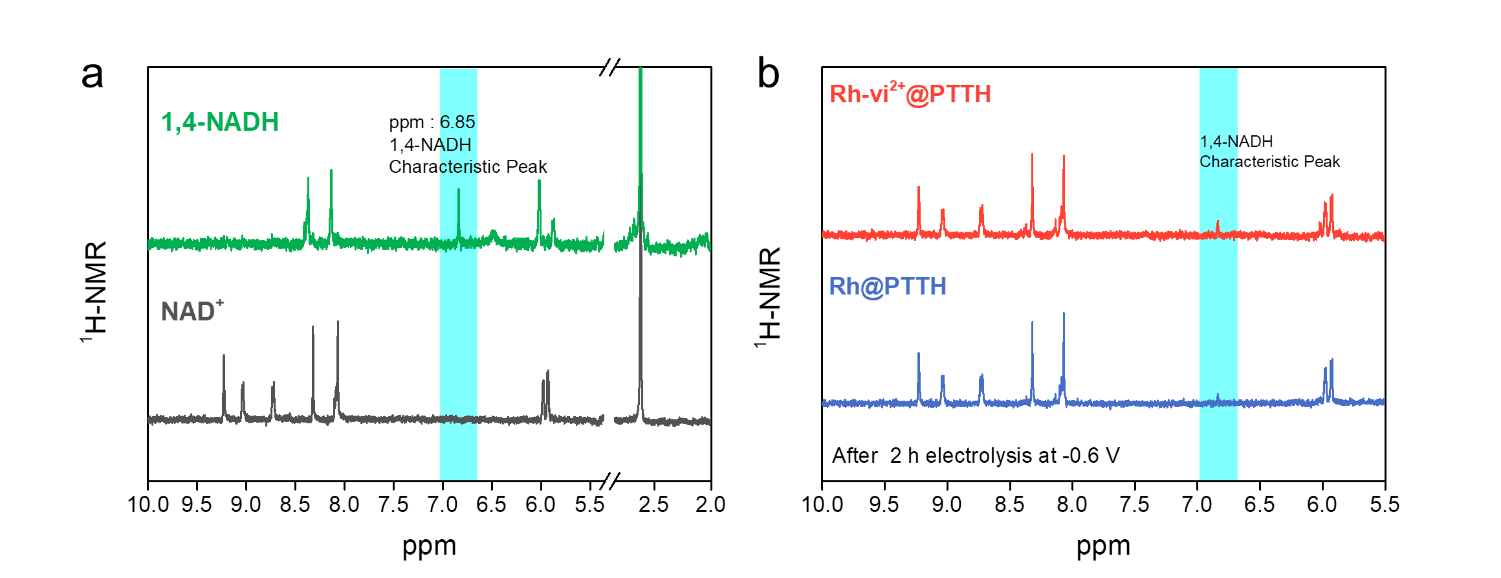
**

**Figure S39**. (a) ^1^H-NMR spectra of standard commercial NAD^+^ and 1,4-NADH samples. (b) The ^1^H-NMR spectra for post-reaction electrolysis of **Rh@PTTH** and **Rh-vi^2+^@PTTH** PEC NADH regeneration systems. Electrolytes: 0.1 M PBS 7 + 0.5 mM NAD^+^ (O_2_-depleted) after 2 hours of electrolysis at 0.0 V vs. RHE.


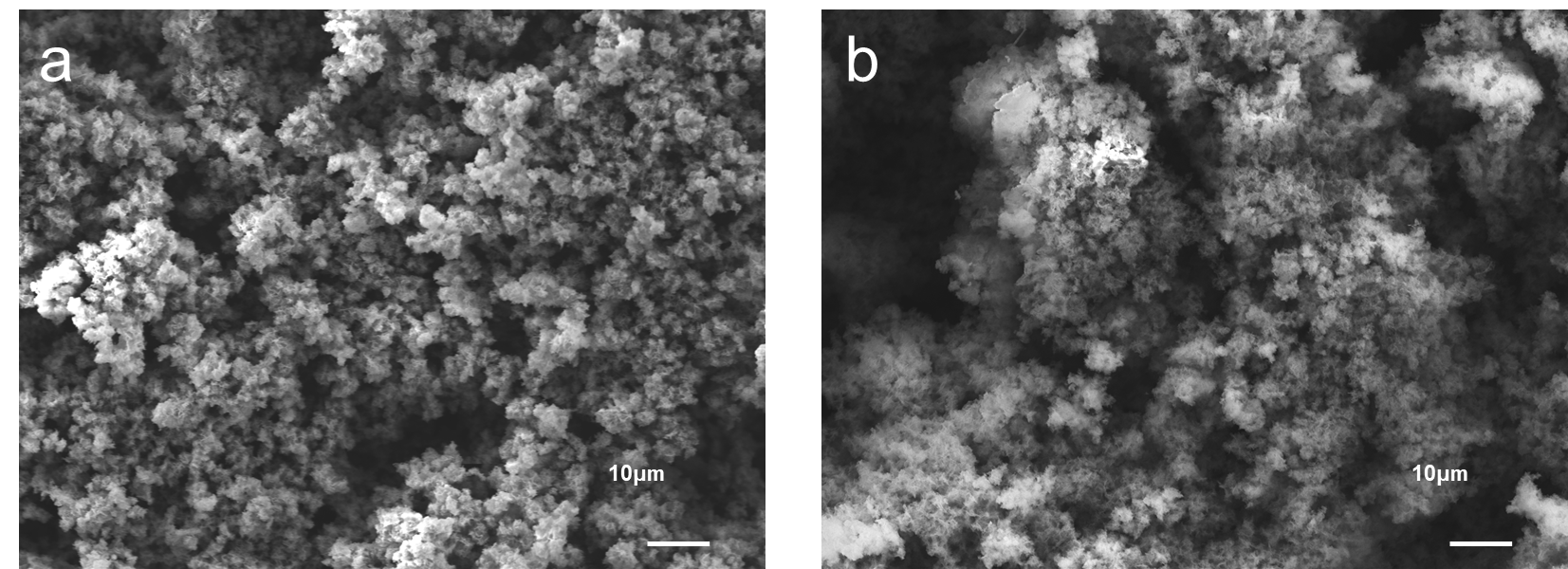


**Figure S40**. SEM images of (a) **Rh@PTTH** photocathode and (b) **Rh-vi^2+^@PTTH** photocathode after 2 hours of continuous electrolysis for NADH regeneration at 0.1 V vs. RHE.


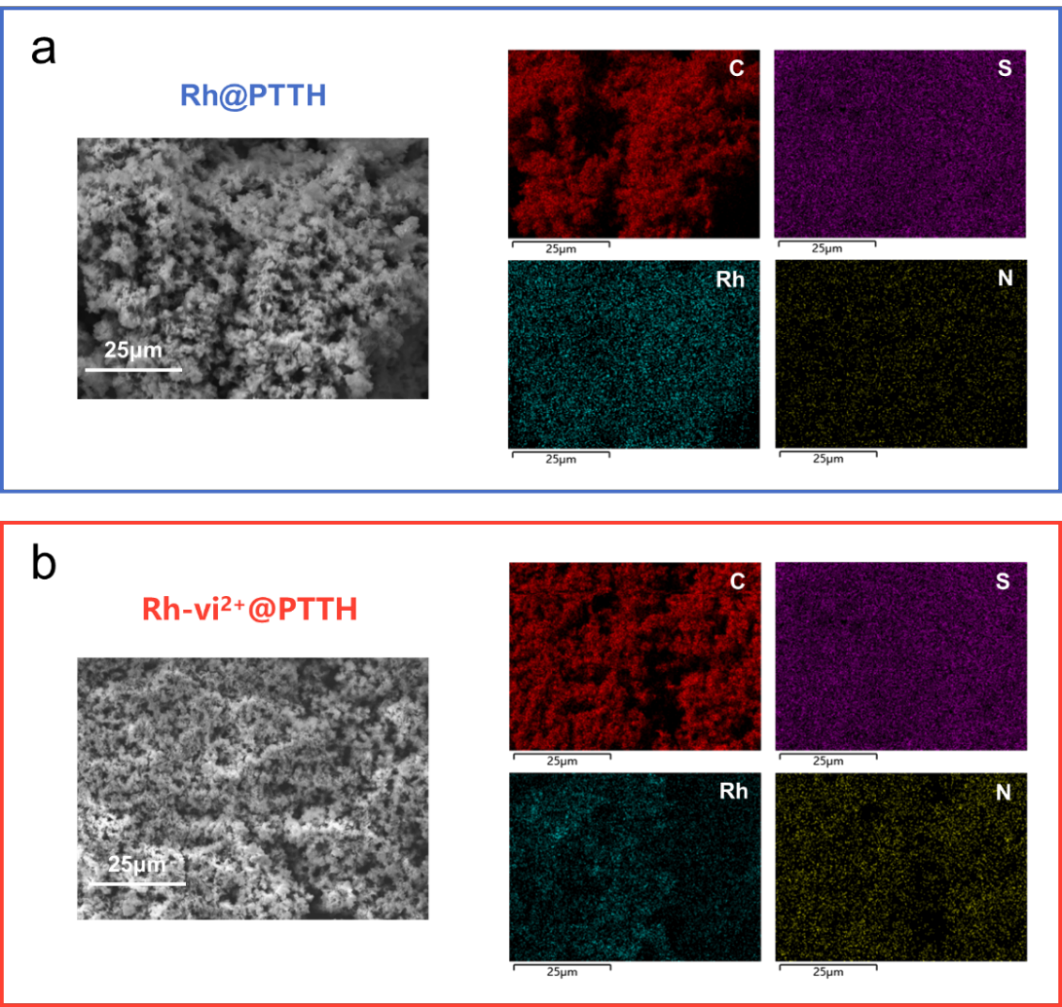


**Figure S41**. SEM images and corresponding EDS elemental mappings for select regions of (a) **Rh@PTTH** and (b) **Rh-vi^2+^@PTTH** photocathode films after NADH regeneration (2 hours of continuous electrolysis) at 0.1 V vs. RHE.

**
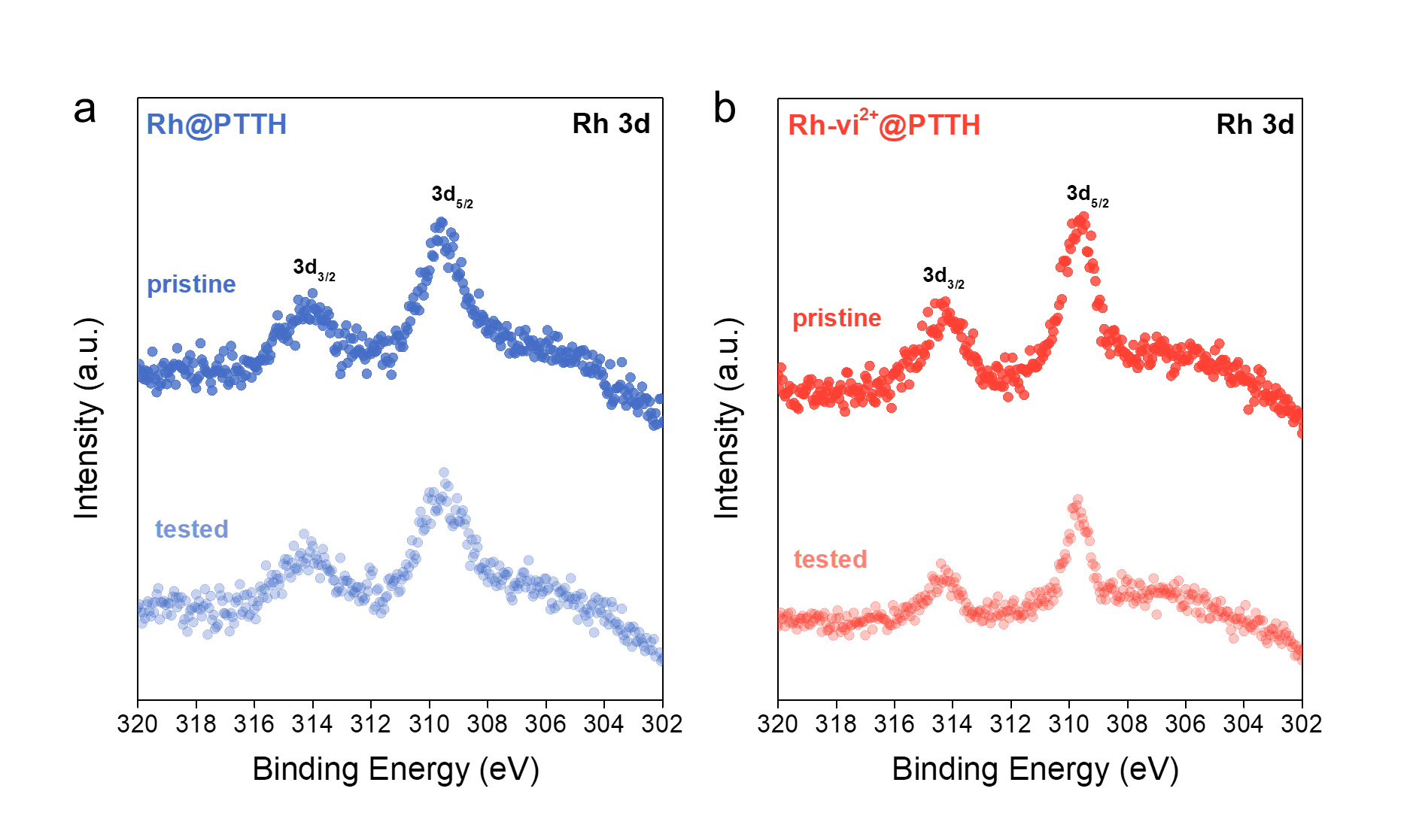
**

**Figure S42**. Comparison of XPS spectra of Rh 3d between before reaction and after 2 hours of photo-electrolysis reaction (a) **Rh@PTTH** and (b) **Rh-vi^2+^@PTTH**.


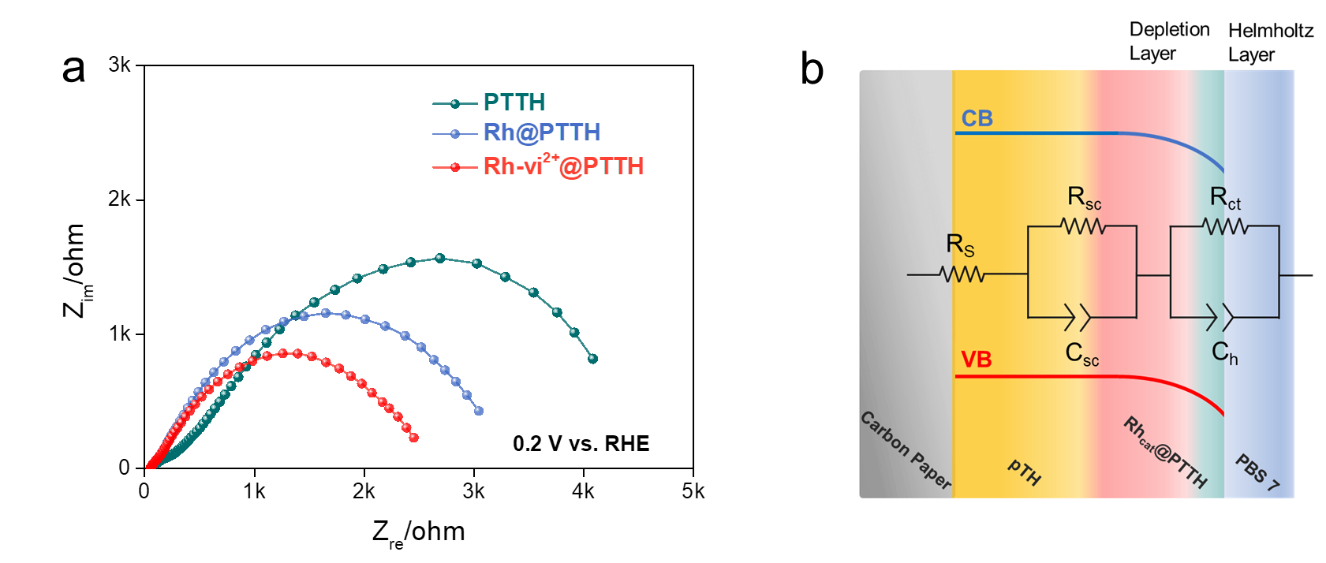


**Figure S43**. (a) Nyquist plots of PEIS measurements in the frequency range of 0.1 to 100 kHz with an AC amplitude (5 mV) under the same illumination and electrolyte conditions. (b) The equivalent circuit of Rh catalyst-modified **PTTH** semiconductor photocathodes.


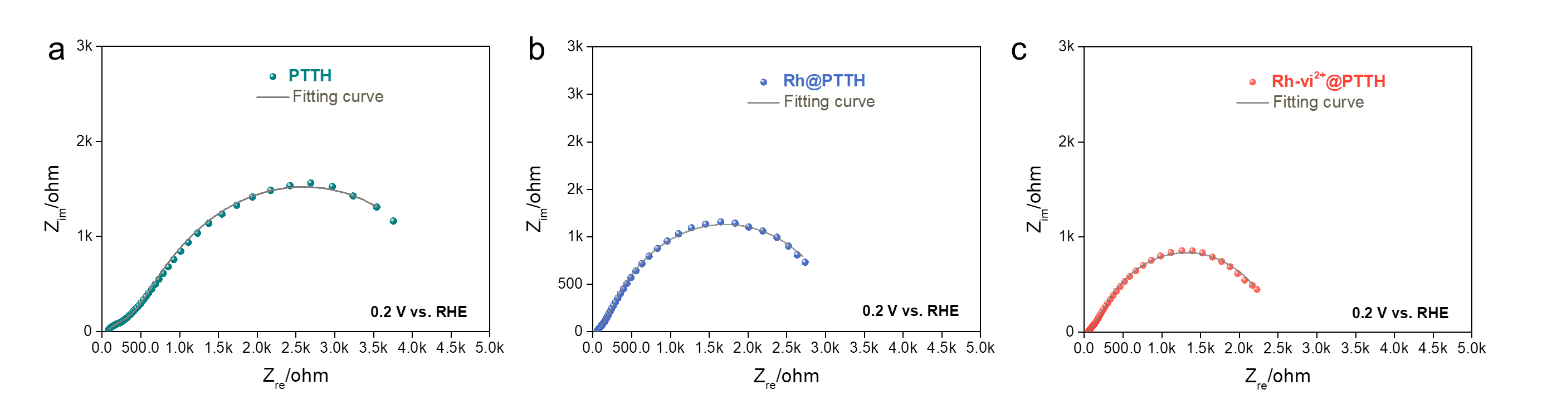


**Figure S44**. Nyquist plots from PEIS experiments of (a) **PTTH** (b) **Rh@PTTH** and (c) **Rh-vi^2+^@PTTH**. (Gray solid lines represent the fitting curve according to the equivalent circuit, respectively).


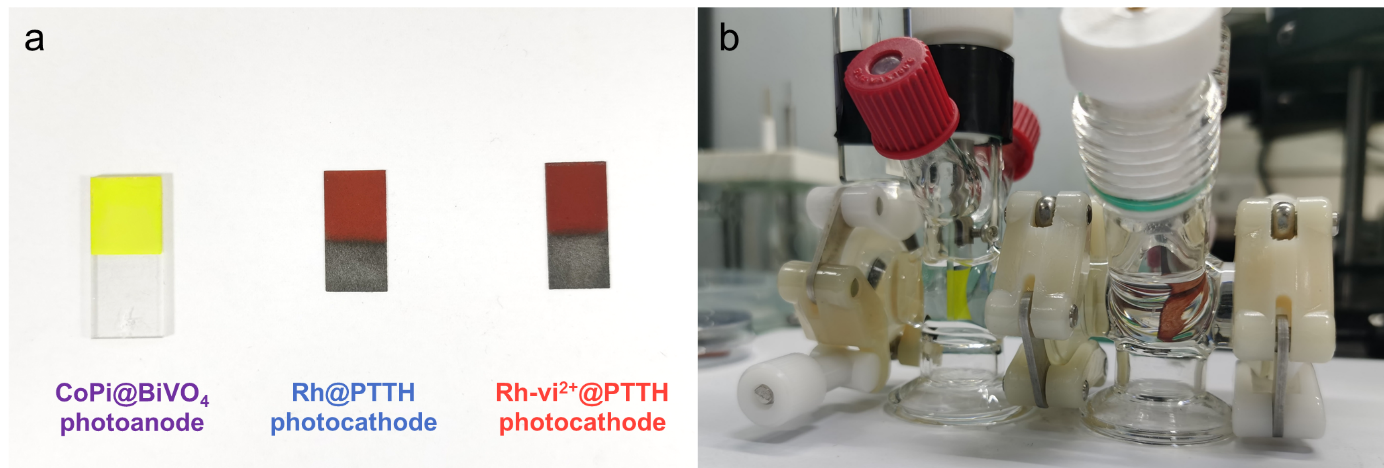


**Figure S45**. (a) Digital photo of prepared **photoanode** (left) and **PTTH-based photocathodes** (right). (b) Digital photo of unbiased two-electrode tandem device for PEC NADH regeneration.

**Figure S46**. LSV curves of **BiVO_4_** and **CoPi@BiVO_4_** photoanodes in 0.1 M PBS (pH 7) under simulated solar illumination with an AM 1.5G filter (100 mW cm^−2^) at a scan rate of 20 mV s^−1^. For realizing the application in unbiased two tandem cells.


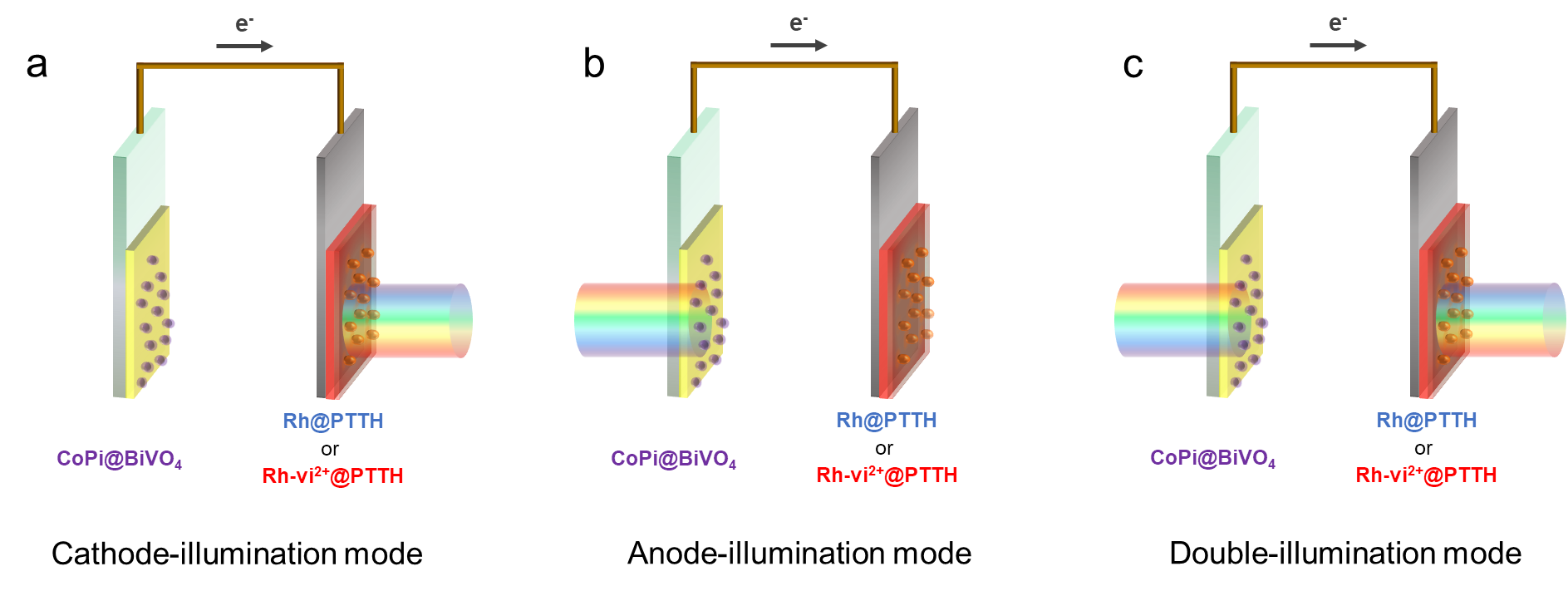


**Figure S47**. The tandem PEC configuration with different illumination patterns from the cathode side (a), anode side (b), and both sides (c).


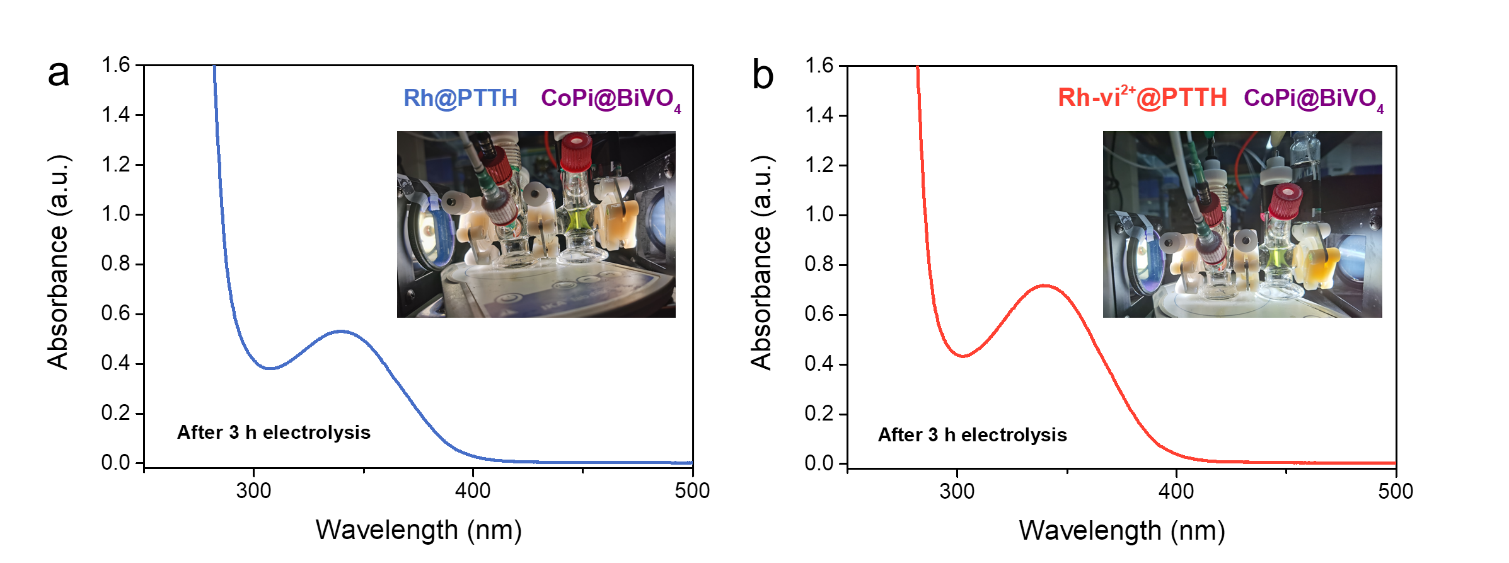


**Figure S48**. UV-vis absorption spectra of the electrolyte in the cathode chamber after 3 hours of unbiased PEC chronoamperometry electrolysis coupled with the **CoPi@BiVO_4_** photoanode for different photocathodes (a) **Rh@PTTH** and (b) **Rh-vi^2+^@PTTH**. (Inset: digital photos of H-type cells under the unbiased PEC operating condition)


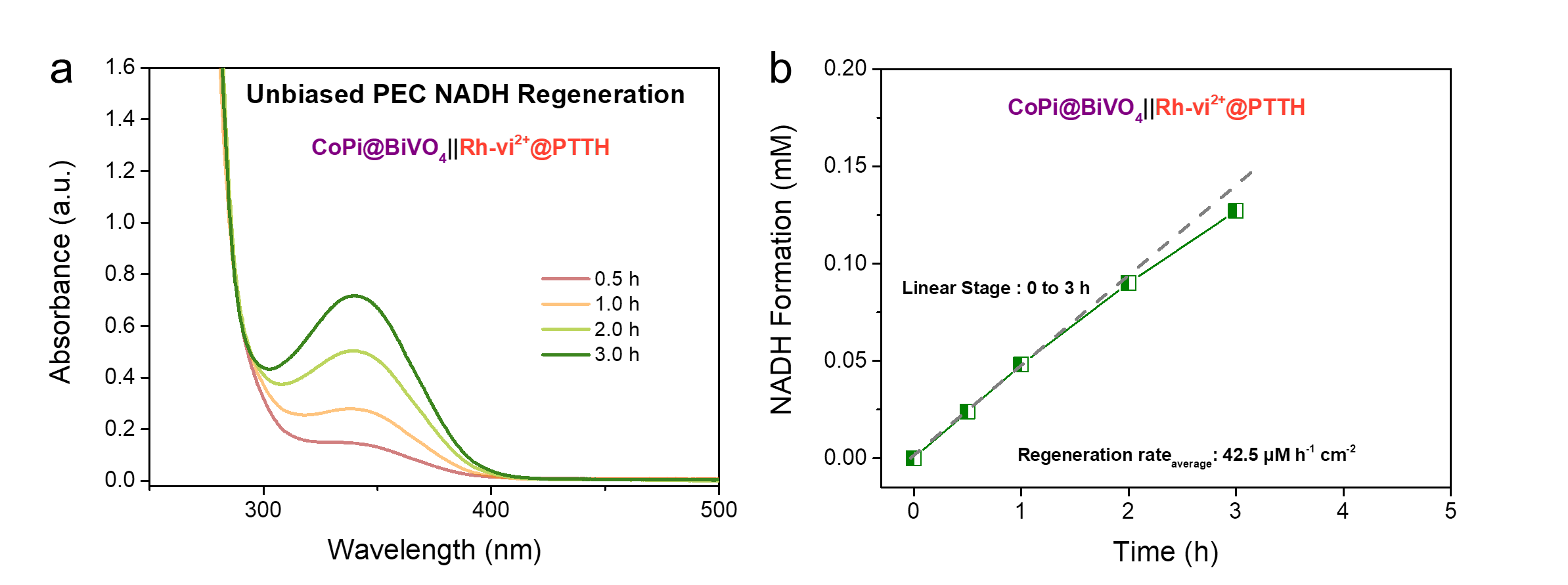


**Figure S49**. (a) UV-vis absorption spectra of the electrolyte after different times of unbiased PEC NADH regeneration for the **CoPi@BiVO_4_||Rh-vi^2+^@PTTH** tandem system. (b) Relationship between the amount of NADH formation and reaction time. (corresponding average regeneration rate is about 42.5 μM h^−1^ cm^−2^)


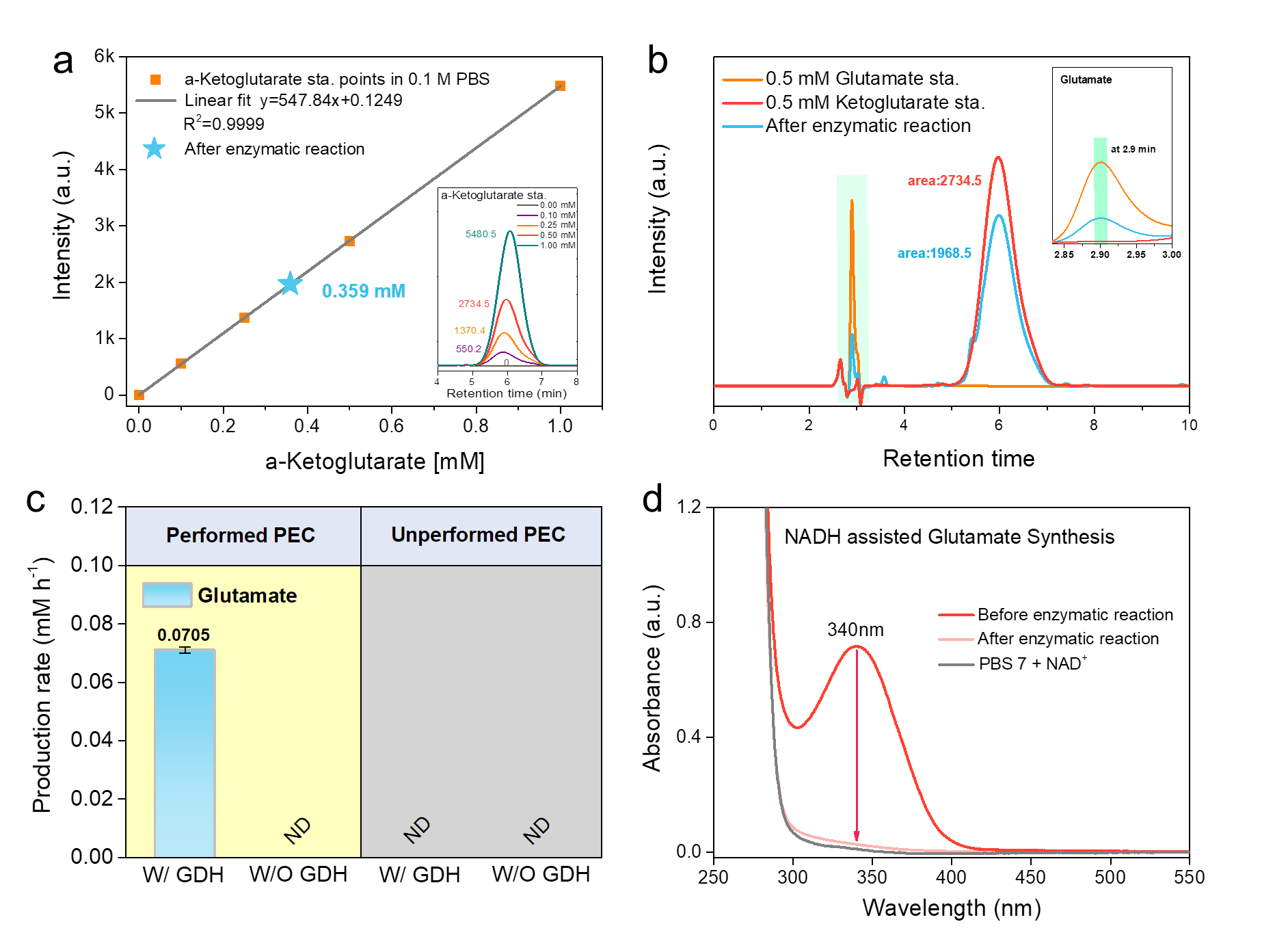


**Figure S50**. (a) The calibration curve of standard a-ketoglutarate, and the blue star point represent the remaining a-ketoglutarate after enzymatic reaction. (Inset: HPLC curves of a-ketoglutarate) (b) HPLC curves of standard glutamate (0.5 mM), ketoglutarate (0.5 mM) and after enzymatic reaction (Inset: enlarge of retention time at 2.9 min, where glutamate was detected) (c) Glutamate production of the PEC-enzyme coupled catalytic system assisted by in-situ NADH generated through unbiased **CoPi@BiVO_4_||Rh-vi^2+^@PTTH** electrolysis (3 hours). W/: With, W/O: Without, ND: not detected. (d) UV-vis absorption at 340 nm before and after glutamate dehydrogenase-involved enzymatic reaction.

# Supplementary Tables

**Table S1.** Summary sheet of binding energies in XPS measurement


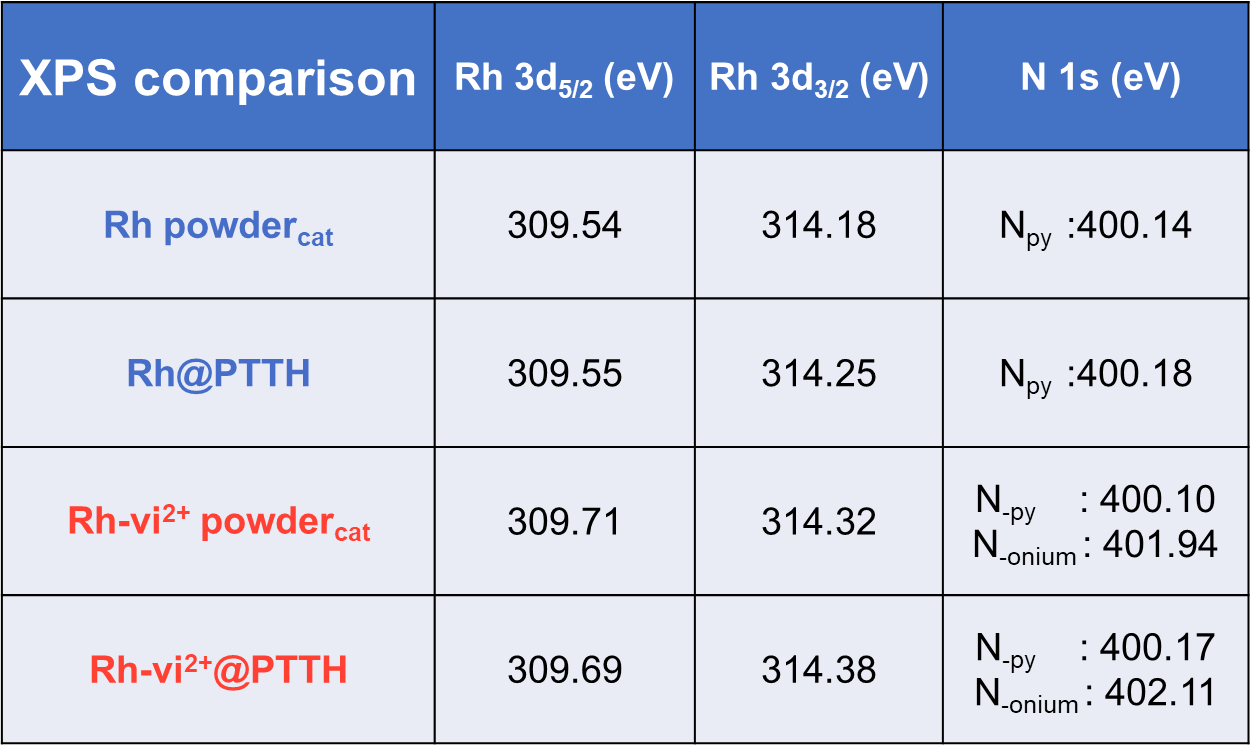


**Table S2.** Comparison of the performance of photocathodes in this work with other reported photocathodes or tandem systems for PEC NADH regeneration reaction

| Photocathode or  Tandem device | Electrolyte | [NAD^+^] | [Rh(Cp*)(bpy)] | Yield of NADH | TOF | Ref. |
| --- | --- | --- | --- | --- | --- | --- |
| **Rh-vi^2+^@PTTH** | PBS, pH 7.0 | 0.5 mM | 2.37 × 10^−7^ mol cm^−2^ immobilized | 16.0% at  0.0 V_RHE_ in 2 h | 168.4 h^−1^ | This Work |
| **Rh@PTTH** | PBS, pH 7.0 | 0.5 mM | 2.40 × 10^−7^ mol cm^−2^ immobilized | 11.4% at  0.0 V_RHE_ in 2 h | 119.2 h^−1^ | This Work |
| **CoPi@BiVO_4_\|\|**  **Rh-vi^2+^@PTTH** | PBS, pH 7.0 | 0.5 mM | 2.37 × 10^−7^ mol cm^−2^ immobilized | 25.5% without bias  in 3 h | 179.3 h^−1^ | This Work |
| **CoPi@BiVO_4_\|\|**  **Rh@PTTH** | PBS, pH 7.0 | 0.5 mM | 2.40 × 10^−7^ mol cm^−2^ immobilized | 18.6% without bias  in 3 h | 129.1 h^−1^ | This Work |
| pTTh | PBS, pH 6.8 | 3.0 mM | 0.25 mM  in solution | 93.3% at  0.1 V_RHE_ in 3 h | 3.73 h^−1^ | ^14^ |
| n^+^p-SiNW | PBS, pH 7.2 | 1.0 mM | 0.25 mM  in solution | 6.0% at  0.2 V_RHE_ in 1 h | 0.24 h^−1^ ***^a^*** | ^16^ |
| Black Si | PBS, pH 7.5 | 1.0 mM | 0.25 mM  in solution | 13.2% at  0 V_Hg/HgO_ in 2 h | 0.26 h^−1^ ***^a^*** | ^17^ |
| g-C_3_N_4_/graphene hybrid | PBS, pH 7.4 | 1.0 mM | 0.25 mM  in solution | 19.0% at  −0.9 V_Ag/AgCl_ in 2 h | 0.38 h^−1^ ***^a^*** | ^18^ |
| CoPi@α-Fe_2_O_3_**\|\|**  BiFeO_3_ | PBS, pH 7.0 | 1.0 mM | 0.25 mM  in solution | 70.5% under  1.0 V_applied bias_ in 2 h | 1.41 h^−1^ ***^a^*** | ^19^ |
| CoPi/ITO/3-jn-Si**\|\|**H-SiNW | PBS, pH 7.0 | 1.0 mM | 0.25 mM  in solution | 19.2% under  1.8 V_applied bias_ in 3 h | 0.26 h^−1^ ***^a^*** | ^20^ |
| FeOOH/Fe_2_O_3_**\|\|**  b-Si | PBS, pH 7.5 | 1.0 mM | 0.25mM  in solution | 19.7% under  1.2 V_applied bias_ in 2 h | 0.40 h^−1^ ***^a^*** | ^21^ |
| FeOOH/BiVO_4_**\|\|**  Perovskite**\|\|**CNT | PBS, pH 7.5 | 2.0 mM | 0.5 mM  in solution | 58.0% without bias  in 1 h | 2.32 h^−1^ ***^a^*** | ^22^ |
| FeOOH/BiVO_4_**\|\|**  CIGS/ITO | PBS, pH 7.0 | 1.0 mM | 0.25 mM  in solution | 28.0% without bias  in 1 h | 1.12 h^−1^ ***^a^*** | ^23^ |

***^a^*** Data estimated according to the data reported in the reference.

**Table S3.** Fitting parameters for PEIS measurements


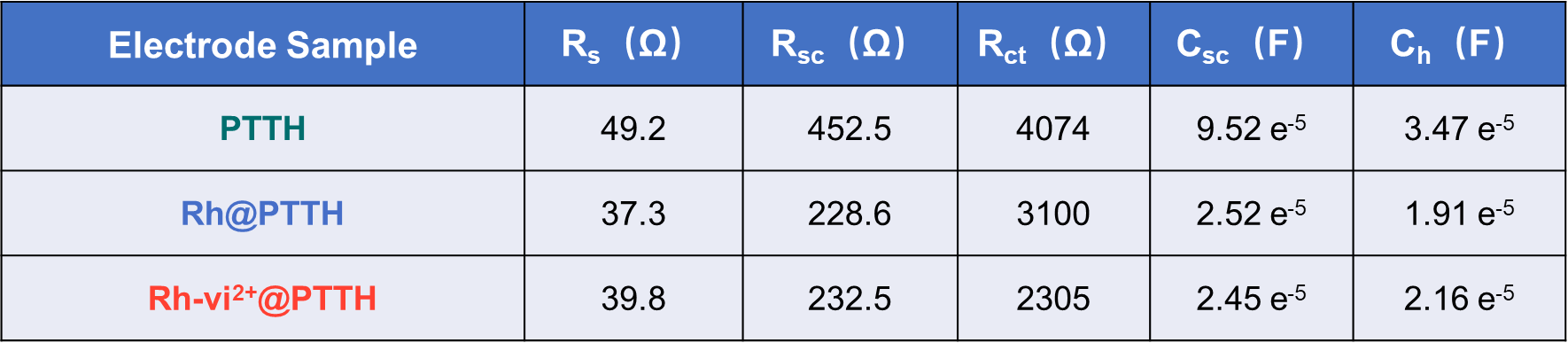


**Table S4.** Film resistivity of two Rh complex-modified electrodes


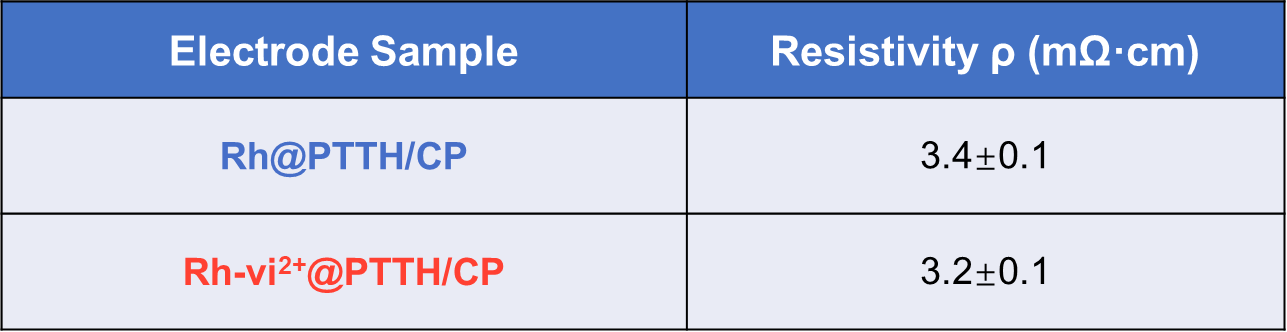


**Table S5.** Biexponential fitting parameters of time-resolved PL decay curves


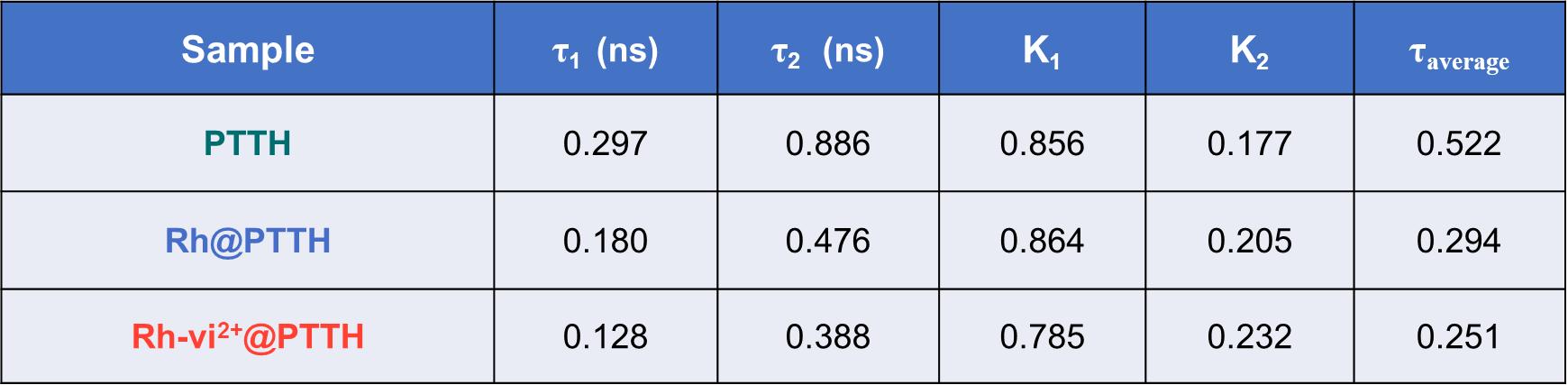


# References

1. M. Chen, F. Liu, Y. Wu, Y. Li, C. Liu, Z. Zhao, P. Zhang, Y. Zhao, L. Sun and F. Li, *Chem. Commun.*, 2024, **60**, 3319-3322.

2. V. Schild, D. van Loyen, H. Dürr, H. Bouas-Laurent, C. Turro, M. Wörner, M. Raj Pokhrel and S. H. Bossmann, *J. Phys. Chem. A,*, 2002, **106**, 9149-9158.

3. C. L. Jones and S. J. Higgins, *J. Mater. Chem.*, 1999, **9**, 865-874.

4. J. Roncali, A. Gorgues and M. Jubault, *Chemistry of Materials*, 1993, **5**, 1456-1464.

5. Y. S. Won, Y. S. Yang, J. H. Kim, J.-H. Ryu, K. K. Kim and S. S. Park, *Energy & Fuels*, 2010, **24**, 3676-3681.

6. Z. Zhao, S. Zhan, L. Feng, C. Liu, M. S. G. Ahlquist, X. Wu, K. Fan, F. Li and L. Sun, *ACS Appl. Mater. Interfaces*, 2021, **13**, 40602-40611.

7. Y. Li, S. Zhan, Z. Deng, M. Chen, Y. Zhao, C. Liu, Z. Zhao, H. Ning, W. Li, F. Li, L. Sun and F. Li, *Journal of Energy Chemistry*, 2024, **93**, 526-537.

8. C. Zachäus, F. F. Abdi, L. M. Peter and R. van de Krol, *Chemical Science*, 2017, **8**, 3712-3719.

9. T. W. Kim and K.-S. Choi, *Science*, 2014, **343**, 990-994.

10. L. Xie, R. Zhang, L. Cui, D. Liu, S. Hao, Y. Ma, G. Du, A. M. Asiri and X. Sun, *Angew. Chem. Int. Ed.*, 2017, **56**, 1064-1068.

11. K. Oka, O. Tsujimura, T. Suga, H. Nishide and B. Winther-Jensen, *Energy & Environmental Science*, 2018, **11**, 1335-1342.

12. J. Šebek, R. Knaanie, B. Albee, E. O. Potma and R. B. Gerber, *J. Phys. Chem. A,*, 2013, **117**, 7442-7452.

13. M. Ates, O. Kuzgun, M. Yildirim and H. Ozkan, *Journal of Polymer Research*, 2020, **27**, 202.

14. N. Li, J. You, L. Huang, H. Zhang, X. Wang, L. He, C. Gong, S. Lin and B. Zhang, *Green Chem.*, 2023, **25**, 5247-5256.

15. S. Tian, S.-M. Lu, T. Liu, F. Liu, C. Feng, X. Zhang, H. Zhang, C. Ding and C. Li, *ChemCatChem*, 2023, **15**, e202300009.

16. E. Lineberry, J. Kim, J. Kim, I. Roh, J.-A. Lin and P. Yang, *Journal of the American Chemical Society*, 2023, **145**, 19508-19512.

17. W. S. Choi, S. H. Lee, J. W. Ko and C. B. Park, *ChemSusChem*, 2016, **9**, 1559-1564.

18. C. Jia, W. Hu, Y. Zhang, C. Teng, Z. Chen and J. Liu, *Inorg. Chem. Front.*, 2020, **7**, 2434-2442.

19. S. K. Kuk, R. K. Singh, D. H. Nam, R. Singh, J.-K. Lee and C. B. Park, *Angew. Chem. Int. Ed.*, 2017, **56**, 3827-3832.

20. E. J. Son, J. W. Ko, S. K. Kuk, H. Choe, S. Lee, J. H. Kim, D. H. Nam, G. M. Ryu, Y. H. Kim and C. B. Park, *Chem. Commun.*, 2016, **52**, 9723-9726.

21. D. H. Nam, G. M. Ryu, S. K. Kuk, D. S. Choi, E. J. Son and C. B. Park, *Applied Catalysis B: Environmental*, 2016, **198**, 311-317.

22. Y. W. Lee, P. Boonmongkolras, E. J. Son, J. Kim, S. H. Lee, S. K. Kuk, J. W. Ko, B. Shin and C. B. Park, *Nat. Commun.*, 2018, **9**, 4208.

23. J. Kim, Y. W. Lee, E.-G. Choi, P. Boonmongkolras, B. W. Jeon, H. Lee, S. T. Kim, S. K. Kuk, Y. H. Kim, B. Shin and C. B. Park, *J. Mater. Chem. A*, 2020, **8**, 8496-8502.
